# Evolution of an Epigenetic Gene Ensemble within the Genus *Anopheles*

**DOI:** 10.1093/gbe/evv041

**Published:** 2015-02-26

**Authors:** Adam M. Jenkins, Marc A.T. Muskavitch

**Affiliations:** ^1^Department of Biology, Boston College; ^2^Discovery Research, Biogen Idec, Cambridge, Massachusetts

**Keywords:** mosquito, epigenetics, comparative genomics, vector genomics, vector biology, malaria

## Abstract

Epigenetic control of gene expression has important implications for the regulation of developmental processes, for mediating homeostasis and responses to the external environment, and for transgenerational inheritance of gene expression patterns. Genes that mediate epigenetic control have been well-characterized in *Drosophila melanogaster*, and we have identified and analyzed an orthologous gene ensemble in *Anopheles gambiae* that comprises 169 orthologs related to a 215-member epigenetic gene ensemble in *D. melanogaster*. We find that this ensemble is highly conserved among anopheline mosquitoes, as we identify only seven gene family expansion/contraction events within the ensemble among 12 mosquito species we have studied within the genus *Anopheles*. Comparative analyses of the epigenetic gene expression across the genera *Drosophila* and *Anopheles* reveal distinct tissue-associated expression patterns in the two genera, but similar temporal expression patterns. The *A. gambiae* complex and *D. melanogaster* subgroup epigenetic gene ensembles exhibit similar evolutionary rates, as assessed by their respective d*N*/d*S* values. These differences in tissue-associated expression patterns, in contrast to similarities in evolutionary rates and temporal expression patterns, may imply that some members of the epigenetic gene ensemble have been redeployed within one or both genera, in comparison to the most recent common ancestor of these two clades. Members of this epigenetic gene ensemble may constitute another set of potential targets for vector control and enable further reductions in the burden of human malaria, by analogy to recent success in development of small molecule antagonists for mammalian epigenetic machinery.

## Introduction

Genome regulation by epigenetic modulation is crucial for many biological processes including development, differentiation, homeostasis, responses to environmental variation, and inheritance of gene expression patterns through generations (Kiefer 2007; Cantone and Fisher 2013; Lunyak and Rosenfeld 2008; Meissner 2010; Greer et al. 2011). Epigenetic control of gene expression through histone acetylation and methylation, and DNA methylation, mediates compaction and decompaction of DNA within euchromatic and heterochromatic chromatin (Guil and Esteller 2009; Greer and Shi 2012). The extent of chromatin condensation is often dependent on the extent of specific posttranslational modifications to histone tails within nucleosomes (Bártová et al. 2008; Zhou et al. 2011). For instance, regulation of developmentally associated genes is controlled by Polycomb- and Trithorax-Group proteins (Schuettengruber et al. 2007; Bracken and Helin 2009; Schwartz and Pirrotta 2007), which have been well-characterized in *Drosophila melanogaster* (Swaminathan et al. 2012; Schuettengruber et al. 2009; Kennison 1995), and other epigenetic modulators. More recent studies have begun to explore the interplay of epigenetic mechanisms with gene family expansion and evolutionary diversification that enables the acquisition of new functions by paralogous gene family members, through divergence in response to selection (Branciamore et al. 2014; Park and Lehner 2014; Sui et al. 2014; Klironomos et al. 2013; Furrow and Feldman 2014; Keller and Yi 2014).

*Drosophila melanogaster* has long constituted a model for studies of epigenetic gene regulation because of the extensive genetic tool set available for the species (Lyko et al. 2006) and because the deep genetics of the Bithorax-Complex and other *Drosophila* developmental genes led to the early discovery of *Polycomb*, *trithorax,* and many other genes that have been shown to be central to epigenetic regulation and modulation of chromatin states through histone modification (Gu and Elgin 2013; Kharchenko et al. 2011; van Bemmel et al. 2013; Schulze and Wallrath 2007; Vermaak and Malik 2009; Swaminathan et al. 2012; Zhou et al. 2013; Foglietti et al. 2006). In contrast, evolution of DNA methylation within the genus *Drosophila* has been investigated based on the presence of a single methyltransferase gene, *Dmnt2*, compared with the multiple DNA methyltransferases found in vertebrates (Marhold et al. 2004). Other studies have implicated DNA methylation and histone modification patterns in the differentiation of caste systems in social insects (Weiner and Toth 2012; Hunt et al. 2013; Elango et al. 2009). Although these studies have often compared genes of interest to orthologs in model or highly studied organisms (e.g., *Homo sapiens*), few comparisons of epigenetic gene ensembles have been conducted among dipteran species, including species within the malaria vector genus *Anopheles* (Arrowsmith et al. 2012; Talbert et al. 2012; Gregoretti et al. 2004). The pan-genomic homology between *D. melanogaster* and *Anopheles gambiae* gene sets has been well-characterized (Zdobnov et al. 2002) and has been leveraged for the identification and curation of orthologous and paralogous genes in *A. gambiae*, as well as for evaluating rates of gene evolution since the divergence of these two dipteran clades (Dottorini et al. 2007; Gregoretti et al. 2004).

We have defined the membership and rates of evolution for the first comprehensive epigenetic gene ensemble to be described in *A. gambiae*, as compared with *D. melanogaster*. We have identified *A. gambiae* genes orthologous to more than 75% of the *D. melanogaster* epigenetic gene ensemble. Our analysis of the *A. gambiae* epigenetic gene ensemble across the genus *Anopheles* reveals very few gene family expansion and contraction events (i.e., four expansion and three contraction events). Different tissue-associated gene expression profiles we detect for members of *A. gambiae* and *D. melanogaster* ensembles imply that a subset of epigenetic genes may have been redeployed since the divergence of these two dipteran clades to mediate differing mechanisms of developmental and behavioral control, coinciding with the existence of many biological differences between these species (i.e., blood feeding, mating behavior). Our analyses provide strong support for the premise that epigenetic control mechanisms are conserved among Anopheline and Drosophilid species, and invite speculation regarding the existence of potentially insecticidable targets among the epigenetic gene ensembles of *A. gambiae* and other vector insects.

## Materials and Methods

### Orthologous Gene Identification

**Fig. 1.—:**
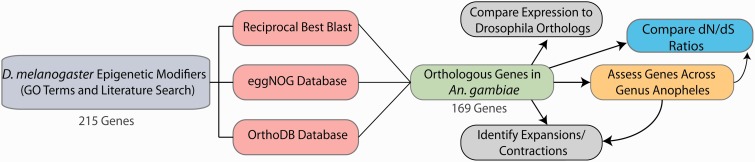
Epigenetic gene set identification and analysis in anopheline species. Chart illustrating the workflow created to identify and analyze homologous epigenetic gene ensembles in *A. gambiae* and other anopheline species. After compiling an epigenetic gene ensemble for *D. melanogaster*, orthologs were identified in *A. gambiae* using Modified Reciprocal Best BLAST, and eggNOG and OrthoDB databases. Temporal expression patterns of orthologous genes were then compared between the two species. Within the genus *Anopheles,* gene number expansions and contractions were identified, and the d*N*/d*S* ratios were calculated and analyzed based on data for multiple members of the *Anopheles* and *Drosophila* clades.

We first defined a comprehensive epigenetic gene ensemble for *D. melanogaster* encompassing genes associated with the Gene Ontology (GO) terms acetyltransferase, ACG/Chrac-complex, beta-heterochromatin, chromatin remodeling, heterochromatin, histone acetylation, histone deacetylation, histone methylation, histone demethylation, histone ubiquitylation, histone deubiquitylation, histone phosphorylation, Ino80 complex, intercalary heterochromatin, Nu4A, nuclear centromeric heterochromatin, nuclear heterochromatin, NuRD complex, RSF complex, Set-N chromatin protein, telomeric heterochromatin, and DNA methylation (Gene Ontology Consortium 2000). This set (table 1) was manually augmented to include genes that were described in primary articles and reviews by Filion et al. (2010), Greer and Shi (2012), van Bemmel et al. (2013), Arrowsmith et al. (2012), Schulze and Wallrath (2007), and Swaminathan et al. (2012). Identification of orthologous genes in *A. gambiae* (fig. 1 and supplementary file S1, Supplementary Material online) was initiated by running TBLASTN using *D. melanogaster* open reading frames as queries against the *A. gambiae* assembly AgamP3.6 from VectorBase (www.vectorbase.org, last accessed March 2015) (Megy et al. 2012), and following this with a modified reciprocal best BLAST (MRBB) analysis. Although strict reciprocal best BLAST identifies 1:1 orthologs, we instead used BLAST to identify initial hits with *E* values less than 1E-10, for each epigenetic modifier gene. These initial hits were used to BLAST against the reciprocal genome, and aligned genes with the highest *E* values were used to define orthologs. This enabled identification of orthologs for genes that have multiple homologs in another species. To further validate putative orthologs, OrthoDB and eggNOG databases were utilized to support MRBB ortholog assignments and to identify potential missed calls (Waterhouse et al. 2013; Powell et al. 2014). To call conclusively an ortholog between *A. gambiae* and *D. melanogaster*, we required that the putative *A. gambiae* ortholog be identified using at least two of the three assessments we applied, that is, MRBB analysis, the eggNOG database and/or the OrthoDB database. In instances in which a putative mosquito ortholog did not satisfy this criterion, and in which we did not therefore “call” an ortholog, a true ortholog may exist in *A. gambiae*, but we will not have called it, based on our stringent criteria.

**Table 1:** Comparison of Epigenetic Gene Ensemble Memberships in *Drosophila melanogaster* and *Anopheles gambiae*

Epigenetic Functional Class Descriptor	Gene Number in *D. melanogaster*	Orthologous Gene Number in *A. gambiae*
Acetylation	26	22
Deacetylation	7	7
Methylation	34	31
Demethylation	7	7
DNA methylation	2	1
Ino80 complex	9	7
ACF complex	4	3
NURF complex	3	3
NuRD complex	6	6
Other complexes	6	6
Heterochromatin	13	8
Centromeric heterochromatin	6	4
Intercalary heterochromatin	5	3
Nuclear heterochromatin	4	3
Other heterochromatin	14	12
Ubiquitylation/phosphorylation	14	12
Set-N proteins and Misc.	55	34

Note.—Gene numbers are based upon orthology between the two species. Functional categorizations are based upon GO terms or known function.

TBLASTN and MRBB analyses were performed among a set of 12 assembled Anopheles genomes (*A. gambiae*, *A. epiroticus*, *A. stephensi*, *A. funestus*, *A. arabiensis*, *A. albimanus*, *A. dirus*, *A. minimus*, *A. quadriannulatus*, *A. atroparvus*, *A. merus*, and *A. farauti*) (Megy et al. 2012), based on the *A. gambiae* epigenetic gene ensemble that we defined using TBLASTN, MRBB, and eggNOG to identify orthologous genes across the genus *Anopheles* (table 1). These ortholog calls were then compared with orthologs identified in the OrthoDB database (Waterhouse et al. 2013). Manual curation was performed for all genes that exhibited inconsistencies among TBLASTN, MRBB, and OrthoDB calls and for which high-depth RNA sequencing data had been produced by Neafsey et al. 2014. We used RNAseq reads for all species (*A. gambiae*, *A. epiroticus*, *A. stephensi*, *A. funestus*, *A. arabiensis*, *A. albimanus*, *A. dirus*, *A. minimus*, *A. quadriannulatus*, *A. atroparvus*, *A. merus*, and *A. farauti*) that are available from SRA accession study PRJNA236161 (Neafsey et al. 2014). Splice junction mapping was performed using TopHat2 (Kim et al. 2013) in relation to the *A. gambiae* P3 genome assembly. A three mismatch maximum was allowed for each read with a maximum -read-edit-dist of three. Gene family expansions that mapped to the *A. gambiae* UNKN chromosome were not designated true expansions/contractions, as these contigs have not been mapped to any chromosome within the initial assembly, and may reflect assembly artifacts rather than genomic differences (Holt et al. 2002; Megy et al. 2012).

### Phylogenetic Assessment and d*N*/d*S* Determination

Phylogenetic relationships were analyzed using DNA sequence alignments and based on maximum likelihood, bootstrapped 100 times, performed by RAxML (Stamatakis 2014). The rate of nonsynonymous substitutions versus the rate of synonymous substitution (or d*N*/d*S* value [Li et al. 1985; Miyata et al. 1980]) for all 1:1 orthologs was determined for the *A. gambiae* complex (comprising *A. gambiae, A. melas, A. merus, A. arabiensis*, and *A*. *quadriannulatus*) based on the ratios calculated using data within the OrthoDB database (Waterhouse et al. 2013). The d*N*/d*S* values for the *D*. *melanogaster* subgroup (*D. melanogaster, D. simulans, D. sechellia, D. yakuba,* and *D. erecta*) were determined by first extracting open reading frame and protein sequences from all *D. melanogaster* OrthoDB orthologs. A coding sequences (CDS)-based alignment was generating using CLUSTAL Omega (Sievers et al. 2011), filtered for at least 60% alignment at any given site using trimAl, and a maximum-likelihood tree was generated using RAxML. The alignment and tree were then submitted to PAML for determination of d*N*/d*S* values by codeml (Yang 2007). Genes that appeared to have saturated d*S* values (>1) or no d*S* value (=0) were not used. The d*N*/d*S* values for single *CC14* paralogs in *A. gambiae* were calculated in comparison to orthologous *D. melanogaster CC14* paralogs using codeml runmode = −2.

### Expression of Epigenetic Modifiers in *A. gambiae* and *D. melanogaster*

Gene expression values were obtained for *A. gambiae* by utilizing RNA sequencing reads from SRA accession number PRJEB5712, and from Pitts et al. (2011). RNA sequencing data sets were aligned using TopHat2 (Kim et al. 2013), as previously described, and FPKM expression values were calculated using CuffDiff (Trapnell et al. 2013; Megy et al. 2012). We utilized the modENCODE expression levels that were given for each gene in FlyBase (www.flybase.org, last accessed March 2015) (St Pierre et al. 2014) to assess *D. melanogaster* gene expression levels. Expression values were grouped among nine distinct life stages, and the average expression level was taken for each life stage. Expression levels were indicated on a scale of 0–6 with the values being 0 = very low/no expression, 1 = low expression, 2 = moderate expression, 3 = moderately high expression, 4 = high expression, 5 = very high expression, and 6 = extremely high expression, in accordance with the expression levels described on FlyBase Release 5.48 (St Pierre et al. 2014).Expression values were then clustered based on the Pearson correlation method using heatmap function in R (R Core Team 2014), for which complete linkage distances and expression classes (high or low expression) were grouped (fig. 4*B* and *C*).

### Principal Component Analysis of Tissue-Specific Gene Expression

Tissue expression values for the epigenetic gene ensembles in *D. melanogaster* and *A. gambiae* were collected from the modENCODE and MozAtlas databases, respectively (Baker et al. 2011; Celniker et al. 2009). Tissues used for principal component analysis (PCA) in both species include carcass, midgut, ovary, testis, head, Malpighian tubules, and salivary gland. Expression values for these tissues were normalized to *Act5C* expression, to correct for potential differences in relative magnitudes of expression in each study. We have chosen *Act5C* for the normalization of gene expression values. Although all genes exhibit some variation in expression across different tissues (Vandesompele et al. 2002), *Act5C* tends to exhibit comparable expression levels for specific tissues of interest, respectively, in both *A. gambiae* and *D. melanogaster* (e.g., *D. melanogaster* gut as compared with *A. gambiae* gut), with the exception of the salivary gland (supplementary file S2, Supplementary Material online), and the *D. melanogaster* ortholog of *Act5C* has been validated as gene for normalization in previous studies (Ponton et al. 2011). PCA was then performed on the relative expression levels of epigenetic gene ensemble members in the tissues previously specified utilizing the prcomp function in R (R Core Team 2014).

## Results

### Defining an Epigenetic Gene Ensemble in A. gambiae

As the basis for defining an epigenetic gene ensemble in *A. gambiae*, we first identified a comprehensive epigenetic gene set in *D. melanogaster*, as described in Materials and Methods (fig. 1). This strategy was motivated by the well-annotated nature of the *Drosophila* genome, the genetic and functional characterizations of many epigenetic modifiers within its genome, and the proximate phylogenetic relationship between these two dipteran species (Lyko et al. 2006; St Pierre et al. 2014; Kharchenko et al. 2011; Zdobnov et al. 2002). We identified 215 epigenetic ensemble genes in *D. melanogaster*, encompassing genes associated with heterochromatin formation and stability, epigenetic complexes, acetylation and deaceytlation, methylation and demethylation, phosphorylation and dephosphorylation, ubiquitylation and deubiquitylation and other epigenetic functions (supplementary file S1, Supplementary Material online), based on comparisons with epigenetic genes in humans (Weng et al. 2012; Arrowsmith et al. 2012). Using MRBB, OrthoDB, and eggNOG, we identified 169 genes in *A. gambiae* (table 1) that are orthologous to members of the 215-member epigenetic gene ensemble that we had defined in *D. melanogaster* (supplementary file S1, Supplementary Material online), as described in Materials and Methods. We required that at least two of the three ortholog identification methods—MRBB, OrthoDB, and/or eggNOG—support the orthologous gene call, in order to define a given gene as being orthologous between the two species. Overall, all three methods positively identified the same ortholog for 146 genes (supplementary file S1, Supplementary Material online), whereas 23 orthologs were identified by only two of the three methods. An ortholog was identified by only one method for each of ten genes, discussed further below. Finally, all three methods failed to detect an ortholog in *A. gambiae* for 36 genes.

Among the 169 orthologous epigenetic gene ensemble members that we define in *A. gambiae*, many complete or nearly complete functional classes are conserved between fruit flies and mosquitoes (table 1). The gene classes within which a plurality of epigenetic modifier genes reside—chromatin acetylation (26 genes in *D. melanogaster*) and chromatin methylation (34 genes in *D. melanogaster*)—are highly conserved, as we identify 22 and 31 orthologous genes for acetylation and methylation classes, respectively, in *A. gambiae*. *Anopheles gambiae* possesses complete sets of orthologs for chromatin deacetylation and demethylation functional classes, including orthologs for all five histone deactylases (Foglietti et al. 2006) and all three arginine-methyltransferases (Boulanger et al. 2004) described in *D. melanogaster*. In total, 68 of the 76 genes that are associated with chromatin methylation/demethylation and chromatin acetylation/deacetylation, including histone demethylases *Kdm4A* and *Kdm4B* and histone methylases *Ash1* and *Ash2*, are conserved between the two species. Among the 28 *D. melanogaster* genes associated with chromatin modifying and remodeling complexes, we identify 25 orthologs in *A. gambiae*. All components of the NuRD and NURF complexes exhibit orthologs in both species, as do nine out of ten other genes involved in the ACF complex and other chromatin-associated complexes. Within the Ino80 complex, seven of nine components exhibit orthologs in both species, as only *CG11970* and *pho* do not exhibit detectable orthologs in *A. gambiae*. All genes in the ubiquitination functional class are conserved, as are five of seven genes within the phosphorylation functional class. Evaluation of heterochromatin-associated genes, that is, centromeric, intercalary and nuclear heterochromatin classes, reveals that *A. gambiae* possesses orthologs for four of six, three of five and three of four *D. melanogaster* genes, respectively, within these three classes.

The multigene Set-N chromatin protein clade in *D. melanogaster* (annotated as *CC*__ in supplementary file S1, Supplementary Material online) (van Bemmel et al. 2013) exhibits the greatest absolute and relative reduction in ortholog number within the epigenetic gene ensemble membership in *A. gambiae*. We are unable to identify *A. gambiae* orthologs for 17 of 40 *Set-N* genes that have been defined in *D. melanogaster*, which accounts for 35% of the total number of genes for which we cannot identify orthologs between these two species. Other *D. melanogaster* genes for which we cannot identify *A. gambiae* orthologs include those encoding two out of the three Ada2a-containing complex components (*Atac1* and *Atac2*) and four other histone modification genes (*BEAF-32*, *Incenp*, *Lpt,* and *msl-1*). Based on our stringent criteria, we also declined to call *A. gambiae* orthologs of six *D. melanogaster* genes involved in heterochromatin modulation: *e(y)3*, *Lhr*, *Pc*, *Prod*, *Su(var)2,* and *Su(var)3–7*.

Based on our criteria for ortholog calling (i.e., at least two of the methods among MRBB, eggNOG, OrthoDB must call the same ortholog), there are ten genes for which only one of these three methods identifies an ortholog in *A. gambiae*: *Borr* (*AGAP0011219*, *AGAP0011220*), *CC34* (*AGAP002753*), *CC35* (*AGAP008006*), *e(y)3* (*AGAP001877*), *HP1b* (*AGAP009444*), *Lpt* (Chromosome 3:18890039–18892840), *Pc* (Chromosome 2:26898592–2757082), *Pcl* (*AGAP003277*), *Su(var)2-HP2* (*AGAP001194*)*,* and *Vig2* (*AGAP013112*). Among these ten genes in *D. melanogaster*, we are able to identify orthologs for seven genes using OrthoDB—*lpt* (7 *Anopheles* species)*, CC34* (4 *Anopheles* species)*, Pc* (17 *Anopheles* species)*, CC35* (18 *Anopheles* species), *e(y)3* (18 *Anopheles* species), *Vig3* (1 *Anopheles* species), and *Hp1b* (14 *Anopheles* species) (supplementary file S3, Supplementary Material online)—among members within the genus *Anopheles*. Our ability to identify *lpt*, *Pc, CC35, e(y)3* and *Hp1b* orthologs in many other *Anopheles* species implies that the putative orthologs for these genes that we have identified in *A. gambiae* are valid, despite not satisfying fully our criteria. The remaining five genes may have true orthologs in *A. gambiae* and all other anophelines assembled to date, but we have not called them based on our stringent criteria. For those fruit fly genes for which we fail to detect orthologs in *A. gambiae* with all three methods (*N* = 36 genes; supplementary file S1, Supplementary Material online), the apparent absence of an ortholog might reflect assembly errors, as complete *A. gambiae* chromosomes are not yet fully assembled (Holt et al. 2002). However, among the 36 genes that yield no ortholog calls in *A. gambiae* using our methods, only two (*msl-1* [13 *Anopheles* species] and *CG11970* [13 *Anopheles* species]) detect putative orthologous genes in other *Anopheles* species in OrthoDB. These findings suggest that the other 34 genes for which we do not detect orthologs in *A. gambiae* may be absent from the *Anopheles* clade.

**Fig. 2.—:**
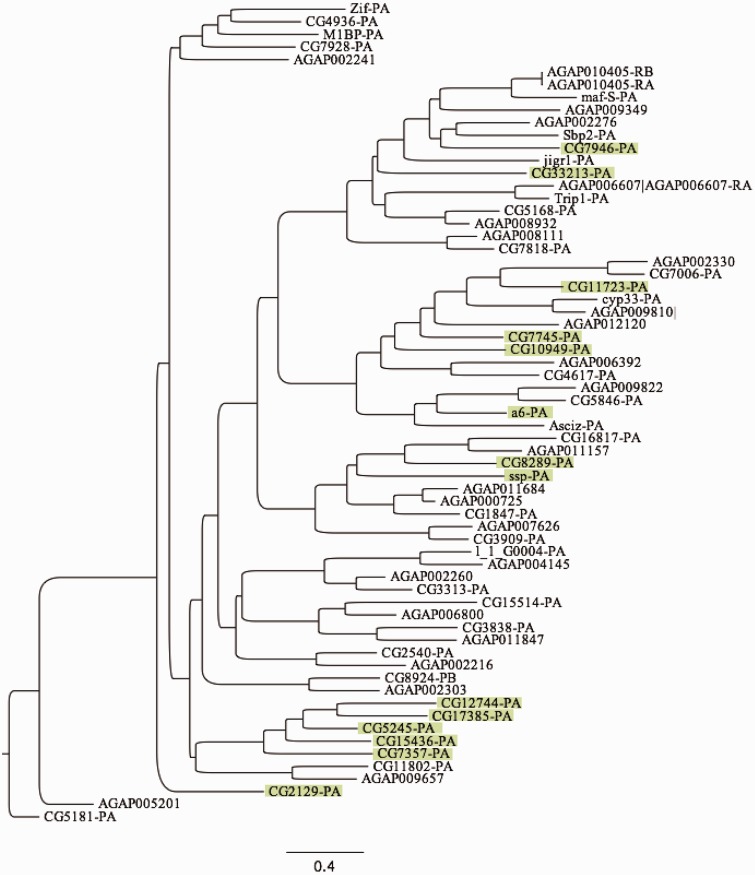
Phylogenetic relationship of Set-N chromatin proteins. Relationships among all *D. melanogaster* and *A. gambiae* Set-N chromatin protein coding-sequences determined using maximum-likelihood (Stamatakis 2014). Green boxes indicate *D. melanogaster* genes for which we do not call an ortholog in *A. gambiae. Anopheles gambiae* genes are depicted by the identifier AGAP and *D. melanogaster* genes are depicted by CG identifier or gene name, if known. For genes with multiple splice variants, isoform RA is represented.

Determining phylogenetic relationships among all *Set-N* gene family member CDS in *D. melanogaster* and orthologous genes in *A. gambiae* by maximum-likelihood using RAxML (Stamatakis 2014) yields inferences regarding differences among species in the evolution of *Set-N* chromatin protein genes (fig. 2). The *D. melanogaster Set-N* chromatin protein gene family includes three related gene clusters for which we do not identify orthologous genes in *A. gambiae*, comprising one group of five *Set-N* genes (*CG15436*, *CG5245*, *CG12744*, *CG17385,* and *CG7357*), a second group of three *Set-N* genes (*CG4936*, *Zif,* and *M1BP*), and a third group of two *Set-N* genes (*ssp* and *CG8289*). Overall, there are 17 *Set-N* genes in *D. melanogaster* for which we do not identify orthologs in *A. gambiae* (fig. 2), consistent with expansion of the *Set-N* gene family in the Brachyceran suborder, as compared with the Nematoceran suborder. Of the 17 *Set-N* genes in *D. melanogaster* for which we do not call an ortholog in *A. gambiae*, we do not detect orthologs for 15 genes among any of the *Anopheles* species genomes annotated within OrthoDB. We do call orthologs for both *CC34* and *CC35* in *Anopheles* species outside of *A. gambiae* (see above).

**Fig. 3.—:**
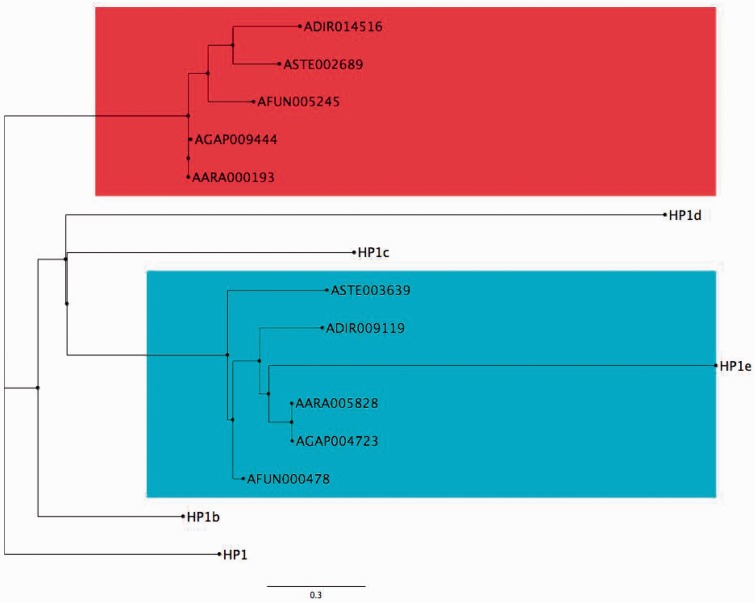
Phylogenetic relationships among *HP1* orthologs in *D. melanogaster* and *A. Gambiae.* Phylogenetic tree of the *HP1* gene family members in *D. melanogaster* (*HP1, HP1b, HP1c, HP1d, HP1e*), *A. gambiae* (AGAP), *A. arabiensis* (AARA), *A. funestus* (AFUN), *A. dirus* (ADIR), and *A. stephensi* (ASTE) calculated using maximum-likelihood method (Stamatakis 2014). The five *Anopheles* species for which genes are depicted exhibit gene number contractions representative of those we observe in all *Anopheles* species analyzed, for the *HP1* gene family. Blue highlight encompasses genes related to *D. melanogaster HP1e*, and red highlight encompasses all other anopheline *HP1* gene family members.

Another gene set that appears to have expanded in the Brachyceran suborder, compared with the Nematoceran suborder, is the *heterochromatin protein-1* (*HP1*) gene family, which has fewer members in *A. gambiae* than in *D. melanogaster*. We identify only two gene family members—*AGAP004723* and *AGAP009444*—in *A. gambiae*, compared with the five *HP1* gene family members—*HP1, HP1b, HP1c, HP1d* (*Rhino*), and *HP1e*—that are present in *D. melanogaster* (fig. 3). In fact, one *HP1b* ortholog (*AGAP009444*) that was identified in *A. gambiae* using MRBB was not supported by either OrthoDB or eggNOG. This reduced *HP-1* gene family membership is also evident among other nematoceran species that span the genus *Anopheles*. Each of the 12 anopheline species we have studied in depth exhibits only two *HP1* gene family members related to the *D. melanogaster HP1* gene family. Comparisons of the expression of orthologous *HP1* family genes in *A. gambiae* and *D. melanogaster* reveal a significant difference in expression patterns of the *D. melanogaster* gene *HP1e* and the *A. gambiae* orthologs *AGAP004723* and *AGAP009444* (supplementary file S2, Supplementary Material online). *HP1e* exhibits little or no expression across all life stages, whereas both *AGAP009444* and *AGAP004723* exhibit significant expression levels among all four life stages/genders assessed, reflective of increased expression of this gene in mosquitoes compared with fruit flies.

### Gene Family Expansions and Contractions across the Genus Anopheles

Among the set of 12 Anopheline species (listed in Materials and Methods) for which high-quality, RNAseq-supported assemblies have been defined (Neafsey et al. 2014), we identify orthologs for all 169 members of the epigenetic gene ensemble we have defined for *A. gambiae* (supplementary file S1, Supplementary Material online). This implies that the dynamic, widespread evolution of the epigenetic gene ensemble that has occurred since the divergence of the suborders *Nematocera* and *Brachycera* appears not to have continued during species divergence within the genus *Anopheles*. In total, seven gene families exhibit expansions or contractions in one or more Anopheline species (table 2). Gene families that include potential paralogs in *A. gambiae*, but for which one of the putative paralogs maps to the *A. gambiae* UNKN chromosome, were neither studied nor shown on table 2, as the UNKN chromosome in the *A. gambiae* genome represents those contigs that were not mapped during initial assembly, and putative gene duplications that map to this “chromosome” may instead constitute assembly artifacts.

**Table 2:** Expansions/Contractions of Epigenetic Modifier Gene Families across the Genus *Anopheles*

D.mel Gene	Gam.	Epi.	Ste.	Fun.	Ara.	Alb.	Dir.	Min.	Qua.	Atr.	Mer.	Far.
*Cap-G*	1	1	1	1	1	1	**2**	1	1	1	1	1
*Parg*	1	1	1	1	1	**0**	1	1	1	1	1	1
*CG18004*	1	1	1	1	1	1	1	1	1	**2**	1	1
*Orc2*	1	1	1	1	1	1	1	1	1	**2**	1	1
*GRO*	2	**1**	2	2	2	2	2	2	2	2	**1**	2
*Effete*	1	1	**1**	**2**	1	**2**	**2**	**2**	1	**2**	1	**2**
*CC14*	2	2	**1**	**1**	2	**1**	**1**	**1**	2	**1**	2	**1**

Note.—Number of orthologous genes that were identified in each of the *Anopheles* species (*Gam.*, *A. gambiae*; *Epi.*, *A. epiroticus*; *Ste.*, *A. stephensi*; *Fun.*, *A. funestus*; *Ara.*, *A. arabiensis*; *Alb.*, *A. albimanus*; *Dir.*, *A. dirus*; *Min.*, *A. minimus*; *Qua.*, *A. quadriannulatus*; *Atr.*, *A. atroparvus*; *Mer.*, *An. merus*; *Far.*, *A. farauti*) corresponding to the original *A. gambiae* ortholgous gene in *D. melanogaster*. Bold ortholog numbers indicate genes that have differing number of orthologs compared to *A. gambiae*.

The *D. melanogaster* genes that exhibit duplications in *A. gambiae*, for which one of the *A. gambiae* orthologous family members maps on the UNKN chromosome, are *Chrac-14*, *Mt2*, and *Wds*. Three anopheline gene families exhibit single species expansions in gene number—*Cap-G* (expanded in *A. dirus*), *CG18004* (expanded in *A. atroparvus*), and *Orc2* (expanded in *A. atroparvus*) (table 2). The *EFF* gene has undergone duplication by retrotransposition in multiple anopheline species, and these duplications have been described elsewhere (Neafsey et al. 2014). We find *CC14* duplications that have arisen through retrotransposition in *A. gambiae*, *A. epiroticus, A. arabiensis*, *A. quadriannulatus*, and *A. merus,* all members of the Pyretophorus Series of Anopheline mosquitoes (fig. 5). Two gene families—*Parg* and *GRO*—exhibit contractions in gene number among the other anopheline species we have studied, relative to *A. gambiae* as *Parg* is contracted in *A. albimanus* and *GRO* is contracted in *A. epiroticus* and *A. merus* (table 2). All other epigenetic gene ensemble members assessed across the genus *Anopheles* exhibit 1:1 orthologous conservation among all 12 anopheline species analyzed.

### Functional and Evolutionary Comparisons of Epigenetic Gene Ensembles

**Fig. 4.—:**
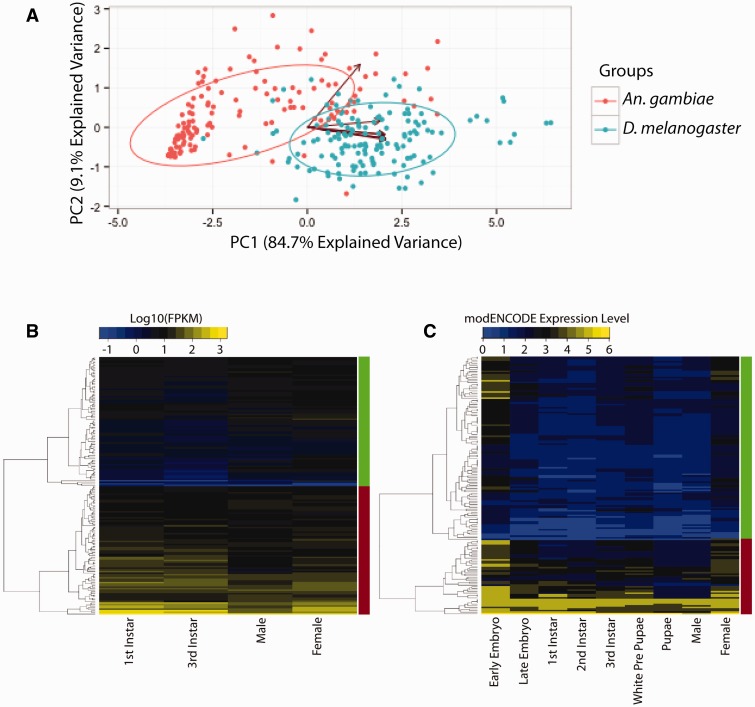
Epigenetic gene ensemble expression in tissues and development. (*A*) PCA (using prcomp function in R; R Core Team 2014) of Log_10_(epigenetic modifier gene expression) across tissues in *D. melanogaster* and *A. gambiae*. Expression values were obtained from modENCODE for *D. melanogaster* and MozAtlas for *A. gambiae* (Baker et al. 2011; Celniker et al. 2009). All values were normalized to *Act5C* to control for potential differences relating to magnitude of expression. Arrows indicate tissue-specific components. Topmost vector (30° off-vertical) represents testis expression, next vector clockwise (85° off-vertical) represents ovary expression, whereas clustered vectors (95° off-vertical) represent carcass, midgut, ovary, head, Malpighian tubules, and salivary gland expression. (*B*) Hierarchical clustering of expression of epigenetic gene ensemble members in *A. gambiae* based on RNA sequencing data across four life stages (mixed gender L1, mixed gender L3, adult male, and adult female) (Jenkins et al. 2014; Jenkins and Muskavitch 2015). Clustering was performed using Pearson correlation with complete linkage distances. Red bars indicate clustering of the “high expression” gene class (84 genes); green bars indicate the “low expression” gene class (85 genes). (*C*) Hierarchical clustering of expression of homologous epigenetic gene ensemble members in *D. melanogaster* based on expression levels identified by modENCODE and listed in FlyBase 5.48 (St Pierre et al. 2014; Celniker et al. 2009). Red bars indicate high expression gene class (50 genes); green bars indicate low expression gene class (119 genes). Comparing heights of same colored bars between panels (*B*) and (*C*) reflects the relative number of genes for each class, in each species.

In order to gain deeper insights into the potential functional similarities and differences between the epigenetic gene ensembles of *A. gambiae* and *D. melanogaster*, we performed a PCA on epigenetic gene expression across comparable tissues in both species (fig. 4*A*). PCA revealed that *A. gambiae* and *D. melanogaster* possess two distinct tissue expression profiles. The two principal components identified account for almost 94% of the variance between the two species. A subset of tissues comprising carcass, midgut, ovary, head, Malpighian tubules, and salivary gland account for 84.7% of the variance, whereas the remaining 9.1% of variance can be attributed predominantly to expression differences within the testis. To evaluate further possible functional differences between the tissue expression profiles in *D. melanogaster* and *A. gambiae*, we compared relative expression levels between the two species for 144 epigenetic genes in seven tissues (supplementary fig. S2, Supplementary Material online). All tissues analyzed exhibited mean increased Log_10_(fold-change in expression values) in *D. melanogaster* between 0.90 and 1.3, with the exception of the testis, which exhibited an increase of only 0.15. The interspecies differences between the fold-change in expression values in testis and all other tissues analyzed were statistically significant using ANOVA (analysis of variance) (*P* < 0.0001).

**Fig. 5.—:**
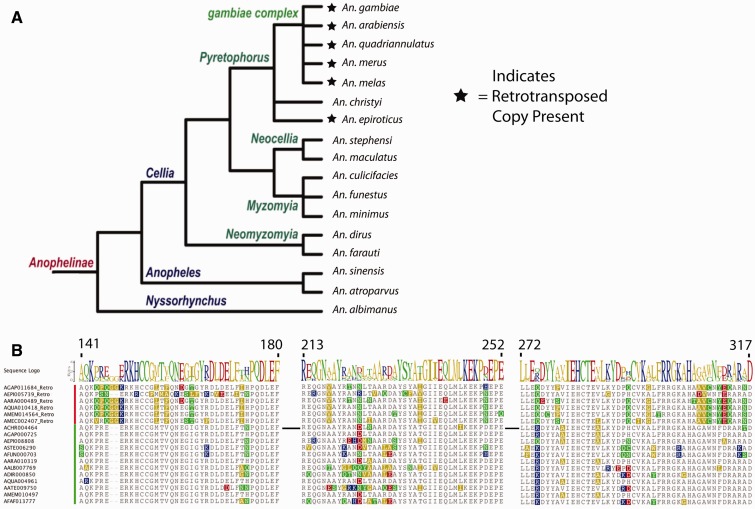
Retrotransposition of *CC14* within the genus *Anopheles.* (*A*) Phylogenetic tree depicting retrotransposition event of *CC14* in the Pyretophorus group. Species that possess the retrotransposed gene are annotated with a star and include *A. gambiae, An. arabiensis, An. quadriannulatus, An. merus, An. melas,* and *A. epiroticus*. We do not detect a retrotransposed copy of *CC14* in *A. christyi*, but this may be due to a suboptimal genome assembly for this species (Neafsey et al. 2014). Dendogram is modified from Neafsey et al. (2013). (*B*) Regions of alignment of retrotransposed and original paralogous *CC14* proteins across *Anopheles*. Retrotransposed genes include “_Retro” at the end of the gene identifier, with red highlight to the left of sequences. Spliced orthologs have a green highlight to left of sequences. Species are given the following identifiers: *A. christyi* (ACHR), *A. gambiae* (AGAP), *An. epiroticus* (AEPI), *A. arabiensis* (AARA), *A. quadriannulatus (*AQUA), *A. merus* (AMEM), *A. stephensi* (ASTE), *A. funestus* (AFUN), *A. albimanus* (ALBI), *A. dirus* (ADIR), *A. atroparvus* (AATE), *A. farauti* (AFAF), *A. melas* (AMEC). Amino acid alignments shown are representations of selected portions of the total open reading frame for each gene, due to the more extensive total lengths of the complete open reading frames. Segments of the open reading frames presented are aa141–180, aa213–252, and aa272–317 in *A. gambiae*.

We next compared developmental expression patterns for orthologous genes between these two species to explore functional conservation between *D. melanogaster* and *A. gambiae* of epigenetic gene ensemble members. Similar analyses have been performed on epigenetic modifier gene ensemble expression profiles in human liver and brain tissue to identify clusters of genes with similar expression patterns (Weng et al. 2012). Hierarchical clustering of gene expression in both species reveals two distinct expression classes: Those genes that possess high expression (red bar) or low expression (green bar) across developmental life stages (fig. 4*B* and *C*). Among these genes within each species, 119 epigenetic genes reside in the same respective high expression (42 genes) or low expression (77 genes) group in mosquitoes and flies, whereas 50 reside in different expression groups in the two species (supplementary fig. S2, Supplementary Material online). Of the 50 genes that exhibit differing expression intensities in these two species, four predominant groups of GO terms are associated with over 75% of the 50 genes—acetylation (14 genes), methylation (ten genes), complexes (six genes) and Set-N chromatin protein genes (eight genes; supplementary fig. S1 and file S2, Supplementary Material online). Four other functional classes—heterochromatin (three genes), phosphorylation (one gene), ubiquitination (two genes), and genes that have no attributable GO term descriptors (six genes)—encompass the remaining genes that exhibit differing expression intensities between *A. gambiae* and *D. melanogaster*.

To assess evolutionary conservation of epigenetic gene ensemble members, and gauge any differences in evolutionary rates, we calculated d*N*/d*S* for each gene within the *A. gambiae* complex and *D. melanogaster* subgroup (supplementary file S4, Supplementary Material online). Direct assessment of respective evolutionary rates is tenable because both the *A. gambiae* complex and *D. melanogaster* subgroup are approximately 5 Myr old (Obbard et al. 2012; Neafsey et al. 2014), enabling estimation of relative evolutionary rates across the same time interval. The average d*N*/d*S* rate (±SEM) for epigenetic genes in the *A. gambiae* complex was 0.1084 (±0.0089) and whereas that for the *D. melanogaster* subgroup was 0.1028 (±0.0068), reflecting the absence of a statistically significant difference in evolutionary rates (*P* = 0.61, *t*-test) (supplementary file S4, Supplementary Material online).

## Discussion

We began this study by assigning 215 genes to the epigenetic gene ensemble of *D. melanogaster* (fig. 1 and supplementary table S1, Supplementary Material online). This ensemble represents approximately 1.5% of the protein-coding genes annotated in the *D. melanogaster* genome (among a total of 13,955 genes; St Pierre et al. 2014). We have defined an even smaller epigenetic gene ensemble in *A. gambiae*. The fact that these limited sets of epigenetic genes are sufficient to control many varied and complex pan-genomic processes encourages the premise that these genes have evolved under strong selective pressure. This premise is supported by low d*N*/d*S* rates we observe for the epigenetic ensemble genes in *D. melanogaster* and *A. gambiae*, as well as the limited gene family expansion and contraction across the genus *Anopheles* that we observe for members of this ensemble. It has been noted that long noncoding RNAs (lncRNA) and microRNAs (miRNAs) have roles in epigenetic regulation and therefore supplement the epigenetic gene ensemble that mediates chromatin modification (Lee 2012; Kim and Nam 2006; Kim 2005; He and Hannon 2004; Nie et al. 2012). The limited epigenetic gene ensemble we define for *A. gambiae* certainly mediates only a portion of the epigenetic control required to ensure a fully functional genome, whereas lncRNAs and miRNAs provide other facets of epigenetic control that we and others are only beginning to elucidate (Mercer and Mattick 2013; Lee 2012; Lv et al. 2013; Ponting et al. 2009).

Some proportion of the selective pressure that appears to constrain evolution of the epigenetic gene ensemble may arise from the oft-noted requirement for epigenetic modifiers to operate within the contexts of multicomponent complexes (Conaway RC and Conaway JW 2009; Schuettengruber et al. 2007).The structural requirements that must be satisfied simultaneously for individual members of such complexes to maintain multiple interactions would constitute one such constraint, which could cause epigenetic genes to be less tolerant of increased mutation rates. The sensitivity of epigenetic machinery to mutation is reflected, in part, by the many alterations in body plan patterning in *Drosophila* that result from alterations in dosages of genes the mediate epigenetic regulation of homeotic gene function (e.g., Polycomb, Trithorax; Schuettengruber et al. 2009, 2007;[Bibr evv041-B76][Bibr evv041-B39][Bibr evv041-B38] Schotta et al. 2002), and the implication that sometimes subtle alterations in epigenetic gene function in a variety of human neoplasias may contribute to oncogenesis (Dawson and Kouzarides 2012; Portela and Esteller 2010). In these and many other instances, a subtle change in the level of function of one member of an epigenetic gene ensemble may contribute to large changes in the developmental or homeostatic landscape of an entire tissue or organism. As this reasoning pertains to the epigenetic gene ensemble in *D. melanogaster*, it will apply to related gene ensembles in other organisms, as well.

For *A. gambiae*, a dipteran of substantial interest due to its propensity to transmit human malaria parasites (Cohuet et al. 2010), we have identified a set of 169 genes that are orthologous to genes within a 215-member epigenetic gene ensemble we have defined in *D. melanogaster* (fig. 1 and supplementary file S1, Supplementary Material online). The conservation rate for epigenetic genes of 79% that we observe between these two species is greater than the 62% interspecies conservation rate observed between the completely annotated genomic-wide protein-coding transcriptomes of *An*. *gambiae* and *D. melanogaster* (Zdobnov et al. 2002). Determination of genome-wide coding transcriptome conservation based on comparisons between *A. gambiae* and each of the other anopheline species we have analyzed yields an average of 99.1% 1:1 orthologous gene number conservation for the 11 pairwise *Anopheles* species comparisons we have completed (see table 2), including only seven instances of epigenetic gene family expansion or contractions across the genus (table 2). Two species (*A. arabiensis* and *A. quadriannulatus*) exhibit 100% 1:1 gene number conservation of the epigenetic gene ensemble when compared with *A. gambiae*. None of the other 11 species compared with *A. gambiae* possesses less than 97.6% 1:1 gene number conservation for the epigenetic gene ensemble. This lowest conservation was observed between *A. gambiae* and *A. atroparvus*, one of the most divergent species pairs among those we have analyzed (Neafsey et al. 2014). The most divergent species pair analyzed—*A. gambiae* and *A. albimanus*—exhibits 1:1 gene number conservation of 98.8%. The greater rates for epigenetic gene conservation that we observe, compared with those observed for the genome-wide protein-coding transcriptomes, provide further evidence of the action of selective pressure on epigenetic gene ensembles since the divergence of Brachycera and Nematocera, as well as during divergence among Anopheline species. Furthermore, the limited number of paralogs (four in total; *Cap-G* in *A. dirus*, *CG18004* and *Orc2* in *A. atroparvus*, and *CC14* within the Pyretophorus class) that we detect within the epigenetic gene ensembles (table 1) that we define among the anopheline species analyzed implies that the composition of this gene ensemble among these species is relatively stable, as reflected by a nearly constant gene membership. Comparison of the epigenetic gene ensemble membership on the basis of copy number constitutes one measure of the consistency of evolutionary pressure that bears on this gene ensemble. Another useful measure for gauging evolutionary pressure on a given gene set is evolutionary rate.

The inference that the epigenetic gene ensemble has been relatively stable as anophelines have diverged is supported by our finding that evolutionary rates within this gene ensemble are similar between the *A. gambiae* complex and the *D. melanogaster* subgroup (supplementary file S4, Supplementary Material online). We observe average epigenetic gene ensemble d*N*/d*S* values of 0.1084 (±0.008990) for the *A. gambiae* complex and 0.1028 (±0.006837) for the *D. melanogaster* subgroup. Both values are indicative of high levels of purifying selection acting on the epigenetic gene ensembles in both species subgroups (Mugal et al. 2014; Gharib and Robinson-Rechavi 2013). The similar evolutionary rates we observe for both taxa, and the infrequent gene family expansion and contraction events we detect, imply that the gene ensemble is evolutionary stable, for the most part. In striking contrast, however, substantial evolution of gene families encoding the Set-N (fig. 2) and HP1 (fig. 3) proteins has occurred through paralogous expansion and contraction within these two insectan clades. In two other instances of rapid evolution, retrotransposition has led to expansion of the *effete* (Neafsey et al. 2014) and *CC14* gene families (this work, see below) among anopheline mosquitoes.

To explore more deeply the functional conservation within the epigenetic gene ensembles in *A. gambiae* and *D. melanogaster*, we investigated the temporal and tissue-specific gene expression patterns of members of the ensembles in these two species. Tissue-specific expression in *D. melanogaster* and *A. gambiae* was compared using PCA (fig. 4*A*). The two species exhibit well-populated but distinct epigenetic gene expression clusters, respectively, based on PCA. This finding is consistent with the inference that many of these epigenetic modifiers are expressed at different levels in specific tissues within the respective species (supplementary fig. S2, Supplementary Material online). On average, *D. melanogaster* exhibits increased epigenetic gene expression levels for all tissues compared with *A. gambiae* gene expression levels, except for the testis, consistent with the findings of our PCA. These differences in expression levels between organisms are analogous to differences observed in epigenetic gene expression for different human cell types (e.g., liver and brain, Weng et al. 2014), suggesting that substantial differences in epigenetic gene expression may be important for cellular distinctions not only between species but also within single species.

Temporal developmental expression patterns for epigenetic ensemble genes in *D. melanogaster* and *A. gambiae* exhibit broad similarity (fig. 4*B* and *C*). A set of 119 *A. gambiae* genes and their *D. melanogaster* orthologs are clustered within comparable high (green blocks, fig. 4*B* and *C*) or low (red blocks, fig. 4*B* and *C*) expression groups in both species, whereas 50 *A. gambiae* and *D. melanogaster* orthologs reside within differing respective expression groups (supplementary fig. S1 and file S2, Supplementary Material online). The GO term classes methylation, acetylation, complex components and Set-N chromatin protein are associated with proteins encoded by 75% of the genes that exhibit differing expression profiles. This may reflect developmentally dynamic redeployment within these species of a subset of epigenetic functions that modulate methylation and/or acetylation, since the divergence of *Brachycera* and *Nematocera*. The broad similarities of temporal expression patterns we observe for most members of the epigenetic gene ensembles in these two Dipteran species are comparable to similarities that have been noted in other closely related species for genome-wide, 1:1 orthologs (e.g., between human and mouse, Huminiecki and Wolfe 2004).

We find that 17 *D. melanogaster* Set-N chromatin proteins do not have identifiable orthologs in *A. gambiae*, representing 42.5% of the total *Set-N* gene set in *D. melanogaster*. When all *Set-N* epigenetic ensemble genes in *D. melanogaster* and *A. gambiae* are compared by maximum likelihood, we find that ten instances of gene multiplication in *D. melanogaster* are not present in *A. gambiae* (green highlights, fig. 2), consistent with the inference that the majority of nonorthologous genes in *D. melanogaster* evolved after divergence from the most recent common ancestor with *A. gambiae*. We observe acquisition of new expression profiles for the *Set-N* paralogs *AGAP000725* and *AGAP011684* in *A. gambiae*, which are orthologous to the SET-N chromatin protein gene *CC14* in *D. melanogaster*. In *A. gambiae*, *AGAP000725* exhibits increased expression across all life-stages compared with *AGAP011684*, which exhibits much lower expression levels (supplementary file S2, Supplementary Material online). These variations in expression may reflect acquisition of qualitatively distinct functions for paralogous genes that have been generated by duplication and divergence within the Nematoceran clade. In fact, a retrotransposition event has contributed to paralogous expansion of the *CC14* gene within the *Set-N* gene family in anophelines (fig. 5*A*). The distinct amino acid profiles we observe within the retrotransposed and original copies (fig. 5*B*) indicate that the two genes may now be under different evolutionary selective pressures. To further explore this inference, we determined the d*N*/d*S* ratios for *AGAP011684* and *AGAP000725*, respectively, as compared with the *D. melanogaster* ortholog *CC14*. The rate of nonsynonymous substitutions (d*S*) was highly saturated (d*S* > 50) for the retrotransposed *AGAP011684*, while being far below saturation for the spliced *AGAP011684* (d*S* < 1). These findings imply that the evolutionary pressures acting on *AGAP011684* are much different than those acting on *AGAP000725*, and they correlate with the high number of amino acid substitutions in the retrotransposed *CC14* ortholog *AGAP011684*, as compared with the lower number of substitutions observed for the spliced *CC14* ortholog *AGAP000725* (fig. 5)

Although five *HP1* gene family members have been annotated in *D. melanogaster*, only two are present in the *An*. *gambiae* genome*.* Based on our phylogenetic analyses, a set of *HP1* genes that is evolutionary orthologous to the *HP1e* gene in *D. melanogaster* (fig. 3, blue highlight) is present in the genus *Anopheles*. A second related set of *HP1*-like genes that we can define among the anophelines (fig. 3, red highlight) is not closely related to any of the *D. melanogaster HP-1* family genes. The predominant expression of *HP1e* in male germline cells in *D. melanogaster* has been proposed to contribute to protection of the male germline genome (Vermaak et al. 2005; Vermaak and Malik 2009). However, the *A. gambiae HP1e* ortholog *AGAP004723* exhibits significantly increased expression in female ovaries, suggesting a function more similar to that of *HP1d* in *D. melanogaster*, which is thought to contribute to protection of the female germline genome (Vermaak et al. 2005; Marinotti et al. 2006; Baker et al. 2011). As previously explored in human and mouse (Huminiecki and Wolfe 2004; Lespinet et al. 2002), intraspecific paralogs often acquire new expression patterns and thereby contribute to evolutionary diversity. This is consistent with the diverse range of expression patterns that members of the *HP1* gene family exhibit in *D. melanogaster*. *HP1d* and *HP1e* exhibit very little to no expression during all life stages, whereas *HP1, HP1b* and *HP1c* exhibit increased expression during some life stages and lower expression during other life stages (supplementary file S2, Supplementary Material online). Both *A. gambiae HP1* gene family orthologs exhibit consistent levels of expression among all life stages, indicating potential functional differences between the orthologous *HP1* genes in these two species. This inference is further supported by differences in temporal expression profiles that we observe between the orthologs *HP1e* and *AGAP004723* (fig. 3). The very limited expression of *HP1e* in fruit flies compared with the increased expression of *AGAP004723* in mosquitoes implies that the mosquito ortholog of fruit fly *HP1e* may have acquired a new function during one or more developmental stages, since divergence from the most recent common ancestor of the suborders *Brachycera* and *Nematocera*.

As the *Set-N* and *HP1* gene families expanded among *Brachycera* and *Nematocera* by duplication and divergence, evolutionary constraints bearing on newly arising members of the gene families may have diminished, allowing paralogous genes to diversify and evolve new functions. This is consistent with the premise that paralogous genes contribute to the genesis of increased genetic diversity by serving as substrates for increased rates of sequence evolution and diversification of gene function (Huminiecki and Wolfe 2004).

Sequence orthology is often invoked as the basis for identification of functionally related genes in *A. gambiae* and *D. melanogaster*. However, such identifications, even when further supported by similar expression profiles, remain inferences until validated by functional genomic analysis. Although many essential genes within Homeobox (HOX) Complexes, and the Polycomb and Trithorax Groups have been shown to be functionally conserved across a range of insects, it is difficult to posit functional conservation without functional genomic data (Schuettengruber et al. 2007, 2009; Kennison 2004). Our findings regarding strong selective pressure on the epigenetic ensembles in both *A. gambiae* and *D. melanogaster*, the relative rarity of gene family expansion/contraction events, and similar temporal gene expression profiles between clades provide strong support for the inference that functionality is also conserved for many of these epigenetic genes. However, admittedly, we do observe differing tissue-specific patterns for some epigenetic gene orthologs in each species. Therefore, conclusive statements regarding functional conservation of orthologs should rest on functional genomic validation, which is available in mosquitoes at present based on RNA interference approaches (Keene et al. 2004; Michel et al. 2005) and may prove feasible through gene editing (e.g., CRISPR technology; Cong et al. 2013) in the future. These approaches to functional validation are particularly important in those instances in which specific epigenetic genes are chosen as potentially druggable targets for insecticide development and vector control.

Due to the rapid evolution of insecticide resistance genes in *Anopheles* mosquitoes (Mitchell et al. 2014; Edi et al. 2014), the identification of additional proteins that may serve as the bases for new vector-targeted control interventions has assumed paramount importance (Zaim and Guillet 2002). In choosing a candidate target gene that encodes an essential catalytic activity that could be inhibited by small molecule antagonists (i.e., potential insecticides), it is important to consider the evolutionary dynamics of putative target genes. A candidate target gene for which the catalytic domain is highly conserved among a very diverse set of insects may be less tolerant of de novo mutations that could confer insecticide resistance. However, an antagonist against a protein that is too broadly conserved may function as an insecticide that kills benign insects as well as vector mosquitoes. Therefore, the ideal such proteins will be those that are conserved among members of a vector insect genus, but diverge within benign insect genera (e.g., *Apis*). This divergence could affect a subset of critical active site residues within an otherwise largely conserved catalytic domain, which would enable identification of vector-selective active site-interacting small molecule antagonists. Alternatively, this divergence could affect regions outside of the catalytic domain, which could be targeted by small molecules that destabilize the target protein or interfere with its interactions with essential protein–protein interaction (PPI) partners. Such proteins could constitute good targets because mutations that arise within a catalytic domain that is highly conserved within the genus and confer insecticide resistance would be difficult to maintain, as they would probably impede wild-type protein function. This premise has begun to be investigated for druggable epigenetic targets in cancer and other diseases (Gomez-Diaz et al. 2012; Arrowsmith et al. 2012; Kishore et al. 2013).

Among the epigenetic gene ensemble members we have characterized, the histone methyltransferase *Su(var)3-9* gene encodes a candidate target within the latter group (i.e., divergence outside of the catalytic domain). This protein has similar epigenetic functions across many species, but exhibits a diverse set of structural differences between species, including gene fusions and refission with other genes (Krauss et al. 2006). Small molecules that target these divergent noncatalytic domains, and diminish protein stability (Bill et al. 2014) or PPIs with critical interaction partners (Ammosova et al. 2012) in vector species, could be designed to reduce cross-reactivity with closely related proteins in benign nonvector species.

A more conventional approach to insecticide development (e.g., larvicides), based on inhibition of epigenetic functions, would involve identification of small molecules selective for mosquito orthologs within epigenetic gene families essential for metamorphic development. Many epigenetic modifiers, most notably the Polycomb Group and Trithorax Group genes (Kennison 1995, 2004; Arrowsmith et al. 2012), have been shown to modulate metamorphic development in *D. melanogaster* and other insects. Members of these gene families could be exploited within *A. gambiae* by developing species-selective larvicides and administering them to habitats in which mosquitoes develop.

Another avenue for species-selective mosquito control based on epigenetic genes could involve the incorporation of anopheline epigenetic functions into Anopheles strains analogous to dominant-lethal sterile-insect strains that have been developed for *Aedes aegypti* (Alphey et al. 2010; Phuc et al. 2007). Given the likely functional conservation of epigenetic genes among multiple mosquito species, and potentially among benign insects as well, the use of mass-administered small molecule antagonists to field habitats may produce substantial die-off among multiple off-target insect species. In contrast, the use of sterile-insect strategies that depend on species-restricted genetic transmission of transgenes that mediate directed misexpression of pleiotropic epigenetic genes, which would lead to developmental lethality or adult sterility, would constitute much more selective approaches to mosquito control.

The application of these conceptual and biochemical approaches, coupled with the identification and further characterization of epigenetic gene ensemble members in anopheline species, will continue to deepen our knowledge of vector genetics and biochemistry, and may enable the development of new vector-targeted insecticidal interventions that will reduce the burdens to human health imposed by malaria and other vector-borne diseases.

## Supplementary Material

Supplementary files S1–S4, figures S1 and S2, and table S1 are available at *Genome Biology and Evolution* online (http://www.gbe.oxfordjournals.org/).

## Acknowledgments

This work was funded by the Biology Department of Boston College. We would also like to thank Robert Waterhouse and Daniel Neafsey for their helpful advise throughout this study and during preparation of the manuscript.

## Supplementary Material

Supplementary Data

## References

[evv041-B1] Alphey L (2010). Sterile-insect methods for control of mosquito-borne diseases: an analysis. Vector-Borne Zoonotic Dis..

[evv041-B2] Ammosova T (2012). Small molecules targeted to a non-catalytic “RVxF” binding site of protein phosphatase-1 inhibit HIV-1. PLoS One.

[evv041-B3] Arrowsmith CH, Bountra C, Fish PV, Lee K, Schapira M (2012). Epigenetic protein families: a new frontier for drug discovery. Nat Rev Drug Discov..

[evv041-B4] Baker DA (2011). A comprehensive gene expression atlas of sex- and tissue-specificity in the malaria vector, *Anopheles gambiae*. BMC Genomics.

[evv041-B5] Bártová E, Krejcí J, Harnicarová A, Galiová G, Kozubek S (2008). Histone modifications and nuclear architecture: a review. J Histochem Cytochem..

[evv041-B6] Bill A (2014). Small molecule-facilitated degradation of ANO1 protein: a new targeting approach for anticancer therapeutics. J Biol Chem..

[evv041-B7] Boulanger M-C (2004). Characterization of the *Drosophila* protein arginine methyltransferases DART1 and DART4. Biochem J..

[evv041-B8] Bracken AP, Helin K (2009). Polycomb group proteins: navigators of lineage pathways led astray in cancer. Nat Rev Cancer..

[evv041-B9] Branciamore S, Rodin AS, Riggs AD, Rodin SN (2014). Enhanced evolution by stochastically variable modification of epigenetic marks in the early embryo. Proc Natl Acad Sci U S A..

[evv041-B10] Cantone I, Fisher AG (2013). Epigenetic programming and reprogramming during development. Nat Struct Mol Biol..

[evv041-B11] Celniker SE (2009). Unlocking the secrets of the genome. Nature.

[evv041-B12] Cohuet A, Harris C, Robert V, Fontenille D (2010). Evolutionary forces on Anopheles: what makes a malaria vector?. Trends Parasitol..

[evv041-B13] Conaway RC, Conaway JW (2009). The INO80 chromatin remodeling complex in transcription, replication and repair. Trends Biochem Sci..

[evv041-B14] Cong L (2013). Multiplex genome engineering using CRISPR/Cas systems. Science.

[evv041-B15] Dawson MA, Kouzarides T (2012). Cancer epigenetics: from mechanism to therapy. Cell.

[evv041-B16] Dottorini T (2007). A genome-wide analysis in *Anopheles gambiae* mosquitoes reveals 46 male accessory gland genes, possible modulators of female behavior. Proc Natl Acad Sci U S A..

[evv041-B17] Edi CV (2014). CYP6 P450 enzymes and ACE-1 duplication produce extreme and multiple insecticide resistance in the malaria mosquito *Anopheles gambiae*. PLoS Genet..

[evv041-B18] Elango N, Hunt BG, Goodisman MAD, Yi SV (2009). DNA methylation is widespread and associated with differential gene expression in castes of the honeybee, *Apis mellifera*. Proc Natl Acad Sci U S A..

[evv041-B100] Filion GJ (2010). Systematic protein location mapping reveals five principal chromatin types in Drosophila cells. Cell.

[evv041-B19] Foglietti C (2006). Dissecting the biological functions of *Drosophila* histone deacetylases by RNA interference and transcriptional profiling. J Biol Chem..

[evv041-B20] Furrow RE, Feldman MW (2014). Genetic variation and the evolution of epigenetic regulation. Evolution (NY).

[evv041-B21] Gene Ontology Consortium (2000). Gene Ontology: tool for the unification of biology. Nature.

[evv041-B22] Gharib WH, Robinson-Rechavi M (2013). The branch-site test of positive selection is surprisingly robust but lacks power under synonymous substitution saturation and variation in GC. Mol Biol Evol..

[evv041-B23] Gomez-Diaz E, Jorda M, Peinado MA, Rivero A (2012). Epigenetics of host–pathogen interactions: the road ahead and the road behind. PLoS Pathog..

[evv041-B24] Greer EL (2011). Transgenerational epigenetic inheritance of longevity in *Caenorhabditis elegans*. Nature.

[evv041-B25] Greer EL, Shi Y (2012). Histone methylation: a dynamic mark in health, disease and inheritance. Nat Rev Genet..

[evv041-B26] Gregoretti IV, Lee Y-M, Goodson HV (2004). Molecular evolution of the histone deacetylase family: functional implications of phylogenetic analysis. J Mol Biol..

[evv041-B27] Gu T, Elgin SCR (2013). Maternal depletion of Piwi, a component of the RNAi system, impacts heterochromatin formation in *Drosophila*. PLoS Genet..

[evv041-B28] Guil S, Esteller M (2009). DNA methylomes, histone codes and miRNAs: tying it all together. Int J Biochem Cell Biol..

[evv041-B29] He L, Hannon GJ (2004). MicroRNAs: small RNAs with a big role in gene regulation. Nat Rev Genet..

[evv041-B30] Holt R (2002). The genome sequence of the malaria mosquito *Anopheles gambiae*. Science.

[evv041-B31] Huminiecki L, Wolfe KH (2004). Divergence of spatial gene expression profiles following species-specific gene duplications in human and mouse. Genome Res..

[evv041-B32] Hunt BG, Glastad KM, Yi SV, Goodisman MAD (2013). Patterning and regulatory associations of DNA methylation are mirrored by histone modifications in insects. Genome Biol Evol..

[evv041-B33] Jenkins AM, Muskavitch MAT (Forthcoming 2015). Crepuscular behavioral variation and profiling of opsin genes in *Anopheles gambiae* and Anopheles stephensi. J Med Entomol..

[evv041-B34] Jenkins AM, Waterhouse RM, Kopin AS, Muskavitch MAT (2014). Long non-coding RNA discovery in *Anopheles gambiae* using deep RNA sequencing. bioarxiv.

[evv041-B35] Keene KM (2004). RNA interference acts as a natural antiviral response to O’nyong-nyong virus (Alphavirus; Togaviridae) infection of *Anopheles gambiae*. Proc Natl Acad Sci U S A..

[evv041-B36] Keller TE, Yi SV (2014). DNA methylation and evolution of duplicate genes. Proc Natl Acad Sci U S A..

[evv041-B37] Kennison JA (1995). The polycomb and trithorax group proteins of *Drosophila*: trans-regulators of homeotic gene function. Annu Rev Genet..

[evv041-B38] Kennison JA (2004). Introduction to Trx-G and Pc-G genes. Methods Enzymol..

[evv041-B39] Kennison JA, Tamkun JW (1988). Dosage-dependent modifiers of polycomb and antennapedia mutations in *Drosophila*. Proc Natl Acad Sci U S A..

[evv041-B40] Kharchenko PV (2011). Comprehensive analysis of the chromatin landscape in *Drosophila melanogaster*. Nature.

[evv041-B41] Kiefer JC (2007). Epigenetics in development. Dev Dyn..

[evv041-B42] Kim D (2013). TopHat2: accurate alignment of transcriptomes in the presence of insertions, deletions and gene fusions. Genome Biol..

[evv041-B43] Kim VN (2005). MicroRNA biogenesis: coordinated cropping and dicing. Nat Rev Mol Cell Biol..

[evv041-B44] Kim VN, Nam J-W (2006). Genomics of microRNA. Trends Genet..

[evv041-B45] Kishore SP, Stiller JW, Deitsch KW (2013). Horizontal gene transfer of epigenetic machinery and evolution of parasitism in the malaria parasite *Plasmodium falciparum* and other apicomplexans. BMC Evol Biol..

[evv041-B46] Klironomos FD, Berg J, Collins S (2013). How epigenetic mutations can affect genetic evolution: model and mechanism. Bioessays.

[evv041-B47] Krauss V, Fassl A, Fiebig P, Patties I, Sass H (2006). The evolution of the histone methyltransferase gene Su(var)3-9 in metazoans includes a fusion with and a re-fission from a functionally unrelated gene. BMC Evol Biol..

[evv041-B48] Lee JT (2012). Epigenetic regulation by long noncoding RNAs. Science.

[evv041-B49] Lespinet O, Wolf YI, Koonin EV, Aravind L (2002). The role of lineage-specific gene family expansion in the evolution of eukaryotes. Genome Res..

[evv041-B50] Li W-H, Wu C-I, Luo C-C (1985). A new method for estimating synonymous and nonsynonymous rates of nucleotide substitution considering the relative likelihood of nucleotide and codon changes. Mol Biol Evol..

[evv041-B51] Lunyak VV, Rosenfeld MG (2008). Epigenetic regulation of stem cell fate. Hum Mol Genet..

[evv041-B52] Lv J (2013). Long non-coding RNA identification over mouse brain development by integrative modeling of chromatin and genomic features. Nucleic Acids Res..

[evv041-B53] Lyko F, Beisel C, Marhold J, Paro R (2006). Epigenetic regulation in *Drosophila*. Curr Top Microbiol Immunol..

[evv041-B54] Marhold J (2004). Conservation of DNA methylation in dipteran insects. Insect Mol Biol..

[evv041-B55] Marinotti O (2006). Genome-wide analysis of gene expression in adult *Anopheles gambiae*. Insect Mol Biol..

[evv041-B56] Megy K (2012). VectorBase: improvements to a bioinformatics resource for invertebrate vector genomics. Nucleic Acids Res..

[evv041-B57] Meissner A (2010). Epigenetic modifications in pluripotent and differentiated cells. Nat Biotechnol..

[evv041-B58] Mercer TR, Mattick JS (2013). Structure and function of long noncoding RNAs in epigenetic regulation. Nat Struct Mol Biol..

[evv041-B59] Michel K, Budd A, Pinto S, Gibson TJ, Kafatos FC (2005). *Anopheles gambiae* SRPN2 facilitates midgut invasion by the malaria parasite *Plasmodium berghei*. EMBO Rep..

[evv041-B60] Mitchell SN (2014). Metabolic and target-site mechanisms combine to confer strong DDT resistance in *Anopheles gambiae*. PLoS One.

[evv041-B61] Miyata T, Yasunaga T, Nishida T (1980). Nucleotide sequence divergence and functional constraint in mRNA evolution. Proc Natl Acad Sci U S A..

[evv041-B62] Mugal CF, Wolf JBW, Kaj I (2014). Why time matters: codon evolution and the temporal dynamics of dN/dS. Mol Biol Evol..

[evv041-B63] Neafsey DE (2013). The evolution of the *Anopheles* 16 genomes project. G3 (Bethesda).

[evv041-B64] Neafsey DE (2014). Mosquito Genomics: Highly evolvable malaria vectors: the genomes of 16 Anopheles mosquitoes. Science.

[evv041-B65] Nie L (2012). Long non-coding RNAs: versatile master regulators of gene expression and crucial players in cancer. Am J Transl Res..

[evv041-B66] Obbard DJ (2012). Estimating divergence dates and substitution rates in the *Drosophila* phylogeny. Mol Biol Evol..

[evv041-B67] Park S, Lehner B (2014). Epigenetic epistatic interactions constrain the evolution of gene expression. Mol Syst Biol..

[evv041-B68] Phuc HK (2007). Late-acting dominant lethal genetic systems and mosquito control. BMC Biol..

[evv041-B69] Pitts RJ, Rinker DC, Jones PL, Rokas A, Zwiebel LJ (2011). Transcriptome profiling of chemosensory appendages in the malaria vector *Anopheles gambiae* reveals tissue- and sex-specific signatures of odor coding. BMC Genomics.

[evv041-B70] Ponting CP, Oliver PL, Reik W (2009). Evolution and functions of long noncoding RNAs. Cell.

[evv041-B71] Ponton F, Chapuis M-P, Pernice M, Sword GA, Simpson SJ (2011). Evaluation of potential reference genes for reverse transcription-qPCR studies of physiological responses in *Drosophila melanogaster*. J Insect Physiol..

[evv041-B72] Portela A, Esteller M (2010). Epigenetic modifications and human disease. Nat Biotechnol..

[evv041-B73] Powell S (2014). eggNOG v4.0: nested orthology inference across 3686 organisms. Nucleic Acids Res..

[evv041-B74] R Core Team (2014). R: a language and environment for statistical computing.

[evv041-B75] Schotta G (2002). Central role of *Drosophila* SU(VAR)3-9 in histone H3-K9 methylation and heterochromatic gene silencing. EMBO J..

[evv041-B76] Schuettengruber B, Chourrout D, Vervoort M, Leblanc B, Cavalli G (2007). Genome regulation by polycomb and trithorax proteins. Cell.

[evv041-B77] Schuettengruber B (2009). Functional anatomy of polycomb and trithorax chromatin landscapes in *Drosophila embryos*. PLoS Biol..

[evv041-B78] Schulze SR, Wallrath LL (2007). Gene regulation by chromatin structure: paradigms established in *Drosophila melanogaster*. Annu Rev Entomol..

[evv041-B79] Schwartz YB, Pirrotta V (2007). Polycomb silencing mechanisms and the management of genomic programmes. Nat Rev Genet..

[evv041-B80] Sievers F (2011). Fast, scalable generation of high-quality protein multiple sequence alignments using Clustal Omega. Mol Syst Biol..

[evv041-B81] St Pierre SE, Ponting L, Stefancsik R, McQuilton P (2014). FlyBase 102—advanced approaches to interrogating FlyBase. Nucleic Acids Res..

[evv041-B82] Stamatakis A (2014). RAxML version 8: a tool for phylogenetic analysis and post-analysis of large phylogenies. Bioinformatics.

[evv041-B83] Sui Y, Li B, Shi J, Chen M (2014). Genomic, regulatory and epigenetic mechanisms underlying duplicated gene evolution in the natural allotetraploid *Oryza minuta*. BMC Genomics.

[evv041-B84] Swaminathan A, Gajan A, Pile LA (2012). Epigenetic regulation of transcription in *Drosophila*. Front Biosci..

[evv041-B85] Talbert PB (2012). A unified phylogeny-based nomenclature for histone variants. Epigenetics Chromatin.

[evv041-B86] Trapnell C (2013). Differential analysis of gene regulation at transcript resolution with RNA-seq. Nat Biotechnol..

[evv041-B87] van Bemmel JG (2013). A network model of the molecular organization of chromatin in *Drosophila*. Mol Cell..

[evv041-B88] Vandesompele J, De Preter K, Poppe B, Van Roy N, De Paepe A (2002). Accurate normalization of real-time quantitative RT -PCR data by geometric averaging of multiple internal control genes. Genome Biol..

[evv041-B89] Vermaak D, Henikoff S, Malik HS (2005). Positive selection drives the evolution of rhino, a member of the heterochromatin protein 1 family in *Drosophila*. PLoS Genet..

[evv041-B90] Vermaak D, Malik HS (2009). Multiple roles for heterochromatin protein 1 genes in *Drosophila*. Annu Rev Genet..

[evv041-B91] Waterhouse RM, Tegenfeldt F, Li J, Zdobnov EM, Kriventseva EV (2013). OrthoDB: a hierarchical catalog of animal, fungal and bacterial orthologs. Nucleic Acids Res..

[evv041-B92] Weiner SA, Toth AL (2012). Epigenetics in social insects: a new direction for understanding the evolution of castes. Genet Res Int..

[evv041-B93] Weng MK (2012). Extensive transcriptional regulation of chromatin modifiers during human neurodevelopment. PLoS One.

[evv041-B94] Weng MK (2014). Lineage-specific regulation of epigenetic modifier genes in human liver and brain. PLoS One.

[evv041-B95] Yang Z (2007). PAML 4: phylogenetic analysis by maximum likelihood. Mol Biol Evol..

[evv041-B96] Zaim M, Guillet P (2002).

[evv041-B97] Zdobnov EM (2002). Comparative genome and proteome analysis of *Anopheles gambiae* and *Drosophila melanogaster*. Science.

[evv041-B98] Zhou Q (2013). The epigenome of evolving *Drosophila* neo-sex chromosomes: dosage compensation and heterochromatin formation. PLoS Biol..

[evv041-B99] Zhou VW, Goren A, Bernstein BE (2011). Charting histone modifications and the functional organization of mammalian genomes. Nat Rev Genet..

