# Supplementary material for: Evolution of an Epigenetic Gene Ensemble within the Genus Anopheles
Source: Genome Biol Evol. 2015 Feb 26;7(3):901–15. doi: 10.1093/gbe/evv041 (PMC5322554; doi:10.1093/gbe/evv041)
Supplement: Supplementary Data [file supp_7_3_901__index.html]

Evolution of an Epigenetic Gene Ensemble within the Genus Anopheles — Evolution of an Epigenetic Gene Ensemble within the Genus Anopheles — Supplementary Data 

# Evolution of an Epigenetic Gene Ensemble within the Genus *Anopheles*

## Supplementary Data

files

**Files in this Data Supplement:**

- Supplementary Data - docx file
- Supplementary Data - docx file
- Supplementary Data - docx file
- Supplementary Data - xlsx file
- Supplementary Data - xlsx file
