# Supplementary material for: Evolution of an Epigenetic Gene Ensemble within the Genus Anopheles
Source: Genome Biol Evol. 2015 Feb 26;7(3):901–15. doi: 10.1093/gbe/evv041 (PMC5322554; doi:10.1093/gbe/evv041)
Supplement: Supplementary Data [file supp_evv041_Supp.File_3.docx]

| **D. melanogaster Species** | **OrthoDB An. species Ortholog** | **VectorBase ID** |
| --- | --- | --- |
|  |  |  |
| *lpt* | An. sinensis | ASIS008867 |
|  | An. atroparvus | AATE001570 |
|  | An. farauti | AFAF020040 |
|  | An. culicifacies | ACUA017774 |
|  | An. maculatus | AMAM002451 |
|  | An. melus | AMEC002139 |
|  | An. merus | AMEM016634 |
|  |  |  |
| *Pcl* | NONE | NONE |
|  |  |  |
| *Borr* | NONE | NONE |
|  |  |  |
| *CC34* | An. darlingi | ADAC009895 |
|  | An. albimanus | AALB007218 |
|  | An. atroparvus | AATE008276 |
|  | An. melus | AMEC0022144 |
|  |  |  |
| *CC35* | An. darlingi | ADAC009487 |
|  | An. darlingi | ADAC009490 |
|  | An. albimanus | AALB000209 |
|  | An. sinensis | ASIS023643 |
|  | An. atroparvus | AATE001759 |
|  | An. farauti | AFAF007976 |
|  | An. dirus | ADIR011026 |
|  | An. funestus | AFUN004961 |
|  | An. funestus | AFUN010641 |
|  | An. minimus | AMIN006260 |
|  | An. culicifacies | ACUA028350 |
|  | An. maculatus | AMAM024072 |
|  | An. stephensi | ASTE006310 |
|  | An. stephensiI | ASTEI01772 |
|  | An. epiroticus | AEPI001913 |
|  | An. christyi | ACHR002040 |
|  | An. melas | AMEC010577 |
|  | An. quadriannulatus | AQUA000657 |
|  | An. arabiensis | AARA005117 |
|  | An. merus | AMEM015589 |
|  | An. gambiae | AGAP008006 |
|  |  |  |
| Su(var)2-HP2 | NONE | NONE |
|  |  |  |
| *Vig2* | An. melas | AMEC017847 |
|  |  |  |
| *e(y)3* | An. darlingi | ADAC009982 |
|  | An. albimanus | AALB008839 |
|  | An. sinensis | ASIS008624 |
|  | An. atroparvus | AATE001014 |
|  | An. farauti | AFAF005930 |
|  | An. dirus | ADIR002679 |
|  | An. funestus | AFUN000012 |
|  | An. minimus | AMIN000446 |
|  | An. culicifacies | ACUA014012 |
|  | An. culicifacies | ACUA018369 |
|  | An. maculatus | AMAM004236 |
|  | An. maculatus | AMAM004298 |
|  | An. stephensi | ASTE009849 |
|  | An. stephensiI | ASTEI08744 |
|  | An. epiroticus | AEPI005299 |
|  | An. christyi | ACHR002198 |
|  | An. christyi | ACHR004013 |
|  | An. melas | AMEC014443 |
|  | An. melas | AMEC015889 |
|  | An. quadriannulatus | AQUA007228 |
|  | An. arabiensis | AARA007050 |
|  | An. merus | AMEM000262 |
|  | An. merus | AMEM014561 |
|  | An. gambiae | AGAP001877 |
|  |  |  |
| *Pc* | An. darlingi | ADAC000737 |
|  | An. albimanus | AALB004691 |
|  | An. sinensis | ASIS017009 |
|  | An. atroparvus | AATE001675 |
|  | An. farauti | AFAF012796 |
|  | An. dirus | ADIR004202 |
|  | An. funestus | AFUN006455 |
|  | An. minimus | AMIN007734 |
|  | An. culicifacies | ACUA021143 |
|  | An. maculatus | AMAM000072 |
|  | An. maculatus | AMAM013483 |
|  | An. stephensi | ASTE008279 |
|  | An. stephensiI | ASTEI02630 |
|  | An. epiroticus | AEPI005099 |
|  | An. christyi | ACHR002035 |
|  | An. melas | AMEC006667 |
|  | An. quadriannulatus | AQUA011669 |
|  | An. arabiensis | AARA007404 |
|  | An. merus | AMEM010196 |
|  |  |  |
| *Hp1b* | An. sinensis | ASIS006742 |
|  | An. sinensis | ASIS006778 |
|  | An. atroparvus | AATE008006 |
|  | An. atroparvus | AATE014613 |
|  | An. farauti | AFAF001046 |
|  | An. farauti | AFAF006518 |
|  | An. dirus | ADIR009119 |
|  | An. dirus | ADIR014516 |
|  | An. funestus | AFUN000478 |
|  | An. funestus | AFUN005245 |
|  | An. minimus | AMIN001830 |
|  | An. minimus | AMIN007463 |
|  | An. maculatus | AMAM011207 |
|  | An. stephensi | ASTE002689 |
|  | An. stephensi | ASTE003639 |
|  | An. stephensiI | ASTEI07376 |
|  | An. epiroticus | AEPI006920 |
|  | An. epiroticus | AEPI011576 |
|  | An. epiroticus | AEPI014483 |
|  | An. christyi | ACHR004649 |
|  | An. christyi | ACHR005990 |
|  | An. quadriannulatus | AQUA009725 |
|  | An. quadriannulatus | AQUA011875 |
|  | An. arabiensis | AARA000193 |
|  | An. arabiensis | AARA005828 |
|  | An. merus | AMEM002096 |
|  | An. gambiae | AGAP004723 |
