# Supplementary material for: Evolution of an Epigenetic Gene Ensemble within the Genus Anopheles
Source: Genome Biol Evol. 2015 Feb 26;7(3):901–15. doi: 10.1093/gbe/evv041 (PMC5322554; doi:10.1093/gbe/evv041)
Supplement: Supplementary Data [file supp_evv041_Supp_Fig_FINAL.docx]

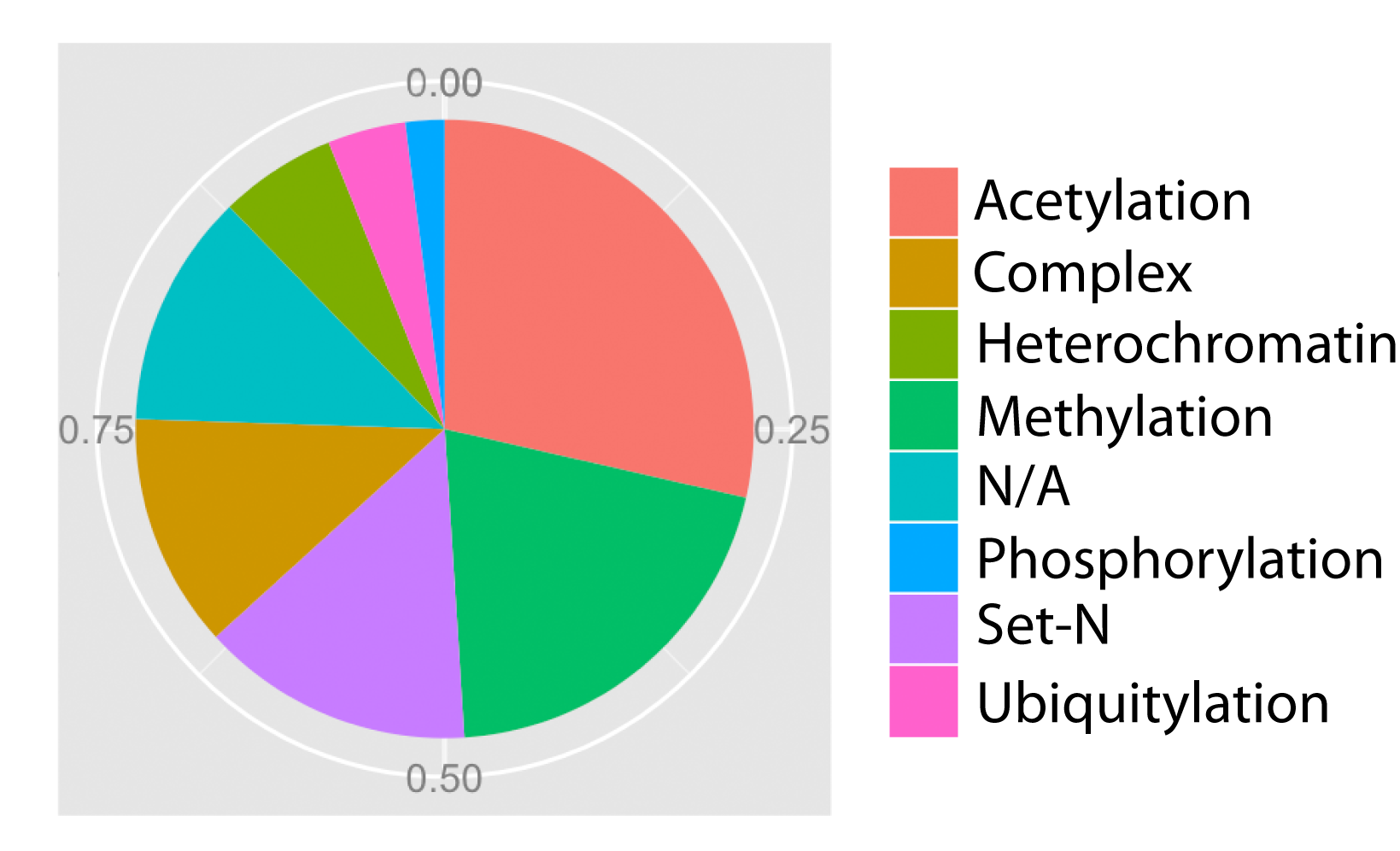


**Supp. Figure 1: GO Terms of Genes with Temporally Unique Expression Profiles Between Species:** Epigenetic genes that were not clustered in either high or low expression classes (red or green bars respectively, Fig. 4B,C) in *D. melanogaster* or *An. gambiae* were grouped based upon GO terms. A total of 50 genes had different expression profiles, of which 75 percent possessed GO terms pertaining to acetylation, methylation, SET-N, or complex components.


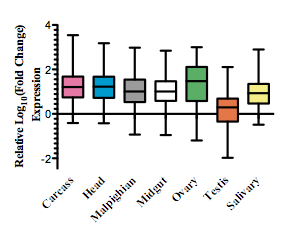


**Supp. Figure 2: Tissue Expression Difference Between *D. melanogaster* and *An. gambiae***: For each tissue used for principal component analysis (Fig. 4A), the relative expression in *D. melanogaster* was compared to the relative expression in *An. gambiae*. Relative expression was calculated by comparing the gene expression to *ACT5C*. Differences in testis compared to the total group of tissues were statistically significant (p-value <0.0001) using ANOVA.
