# Supplementary material for: Evolution of an Epigenetic Gene Ensemble within the Genus Anopheles
Source: Genome Biol Evol. 2015 Feb 26;7(3):901–15. doi: 10.1093/gbe/evv041 (PMC5322554; doi:10.1093/gbe/evv041)
Supplement: Supplementary Data [file supp_evv041_Supp_File_1.docx]

| ***An. gambiae* Orthologous Gene Discovery of *D. melanogaster* Epigenetic Modifiers** | | | | | | | | | |
| --- | --- | --- | --- | --- | --- | --- | --- | --- | --- |
| **^[[1]](#footnote-1)^Dmel Gene Name** | **^[[2]](#footnote-2)^Source** | **CG ID** | **^[[3]](#footnote-3)^FlyBase Description** | **^[[4]](#footnote-4)^GO Term/Description** | **^[[5]](#footnote-5)^An. Gambiae BLAST** | **^[[6]](#footnote-6)^Angam:Dmel RBB** | **^[[7]](#footnote-7)^orthoDB** | **^[[8]](#footnote-8)^eggNOG** | **^[[9]](#footnote-9)^Final An. gam Orthologous Gene** |
| Acf1 | GO | CG1966 | ATP-dependent chromatin assembly factor lg. sub | ACF/Chrac Complex | AGAP000668, AGAP003137 | ++ | ++ | + | AGAP000668 |
| ACT5C | Filion *et al.* 2010 | CG4027 | Actin 5C | Complex Associated | AGAP000651 | + | + | + | AGAP000651 |
| Ada2a | GO | CG43663 | Transcriptional Adaptor 2a | Histone Acetylation | AGAP003109 | + | + | - | AGAP003109 |
| Ada2b | GO | CG9638 | Transcriptional Adaptor 2B | Histone Acetylation | AGAP003109 | - (2nd) | + | + | AGAP003109 |
| Arp5 | GO | CG7940 | Actin-related protein 5 | Ino80 Complex | AGAP004488 | + | + | + | AGAP004488 |
| Arp8 | GO | CG7846 | Actin-related protein 8 | Ino80 Complex | AGAP010671 | + | + | + | AGAP010671 |
| Art1 | GO | CG6554 | Arginine-Methyltransferase 1 | Histone Methylation | AGAP003462 | + | + | + | AGAP003462 |
| Art4 | GO | CG5358 | Arginine-Methyltransferase 4 | Histone Methylation | AGAP003923 | + | + | + | AGAP003923 |
| Art8 | GO | CG16840 | Arginine Methyltransferase | Histone Methylation | AGAP008846 | + | + | + | AGAP008846 |
| Asf1 | Filion *et al.* 2010 | CG9383 | Anti-silencing factor 1 |  | AGAP005920 | + | + | + | AGAP005920 |
| Ash1 | GO | CG8887 | Absemt, small, or homeotic discs 1 | Histone Methylation | AGAP001535 | + | + | + | AGAP001535 |
| Ash2 | GO | CG6677 | Absemt, small, or homeotic discs 2 | Histone Methylation | AGAP003705 | + | + | + | AGAP003705 |
| Atac1 | GO | CG9200 | Ada2a-containing complex component 1 | Histone Acetylation | No Hits | N/A | -(none) | - | - |
| Atac2 | GO | CG10414 | Ada2a-containing complex component 2 | Histone Acetylation | No Hits | N/A | -(none) | - | - |
| Atac3 | GO | CG32343 | Ada2a-containing complex component 3 | Histone Acetylation | AGAP006384 | + | + | + | AGAP006384 |
| Atf-2 | GO | CG44246 | Activating transcription factor 2 | Nuclear Centromeric Heterochromatin | AGAP008416 | + | + | + | AGAP008416 |
| ball | GO | CG6386 | ballchen | Histone phosphorylation | AGAP001933 | + | + | + | AGAP001933 |
| BCD | Filion *et al.* 2010 | CG1034 | Bicoid |  | AGAP004647 | - (Other) | -(none) | - | - |
| BEAF-32 | GO | CG10159 | Boundary element-associated factor of 32Kd | Histone Methylation | No Hits | N/A | -(none) | - | - |
| borr | GO | CG4454 | borealin-related | Histone phosphorylation | No Hits | N/A | -(none) | +(AGAP011219, AGAP011220) | - |
| Bre1 | Swaminathan *et al. 12* | CG10542 |  | Ubiquintase | AGAP007335 | + | + | + | AGAP007335 |
| brm | GO | CG5942 | brahma | Chromatin Remodeling | AGAP010462 | + | + | + | AGAP010462 |
| Bur | GO | CG9242 | burgundy | Histone Deubiquitination | AGAP010139 | + | + | + | AGAP010139 |
| Caf1 | GO | CG4236 | Chromatin Assembly Factor 1 subunit | NuRD Complex | AGAP003228 | + | + | + | AGAP003228 |
| Cap-G | GO | CG34438 |  | Nuclear Heterochromatin | AGAP007568 | + | + | + | AGAP007568 |
| Cbp | Swaminathan *et al. 12* | CG1435 | Sarcoplasmic Calcium-Binding Protein | Acetyltransferase | AGAP007172 | - | +(AGAP001177) | +(AGAP01177) | AGAP001177 |
| CC1 | van Bemmel *et al.* 2013 | CG3313 |  | Set-N Chromatin Protein | AGAP002260 | + | + | + | AGAP002260 |
| CC10 | van Bemmel *et al.* 2013 | CG3909 |  | Set-N Chromatin Protein | AGAP007626 | + | + | + | AGAP007626 |
| CC11 | van Bemmel *et al.* 2013 | CG7818 |  | Set-N Chromatin Protein | AGAP008111 | + | + | + | AGAP008111 |
| CC12 | van Bemmel *et al.* 2013 | CG5846 |  | Set-N Chromatin Protein | AGAP009822 | + | + | + | AGAP009822 |
| CC14 | van Bemmel *et al.* 2013 | CG1847 |  | Set-N Chromatin Protein | AGAP011684, AGAP000725 | ++ | ++ | ++ | AGAP011684, AGAP000725 |
| CC15 | van Bemmel *et al.* 2013 | CG14962 |  | Set-N Chromatin Protein | AGAP012120 | + | + | + | AGAP012120 |
| CC16 | van Bemmel *et al.* 2013 | CG11802 |  | Set-N Chromatin Protein | AGAP009655 | -(other) | + | + | AGAP009657 |
| CC17 | van Bemmel *et al.* 2013 | CG5168 |  | Set-N Chromatin Protein | AGAP008932 | + | + | + | AGAP008932 |
| CC18 | van Bemmel *et al.* 2013 | CG16817 |  | Set-N Chromatin Protein | AGAP011157 | + | + | + | AGAP011157 |
| CC19 | van Bemmel *et al.* 2013 | CG2540 |  | Set-N Chromatin Protein | AGAP002216 | + | + | + | AGAP002216 |
| CC2 (lethal (1) G0004) | van Bemmel *et al.* 2013 | CG11738 | lethal (1) G0004 | Set-N Chromatin Protein | AGAP004145 | + | + | + | AGAP004145 |
| CC20 | van Bemmel *et al.* 2013 | CG3838 |  | Set-N Chromatin Protein | AGAP011847 | + | + | + | AGAP011847 |
| CC21 | van Bemmel *et al.* 2013 | CG12744 |  | Set-N Chromatin Protein | AGAP011759, AGAP003446 | -(other) | -(none) | - | - |
| CC22 | van Bemmel *et al.* 2013 | CG2129 |  | Set-N Chromatin Protein | AGAP003111, AGAP013753, AGAP011655 | -(other) | -(none) | - | - |
| CC23 (a6) | van Bemmel *et al.* 2013 | CG3771 | a6 | Set-N Chromatin Protein | No Hits Found | N/A | -(none) | - | - |
| CC24 | van Bemmel *et al.* 2013 | CG5181 |  | Set-N Chromatin Protein | AGAP005201 | + | + | + | AGAP005201 |
| CC25 | van Bemmel *et al.* 2013 | CG11723 |  | Set-N Chromatin Protein | AGAP009140 | -(other) | -(none) | - | - |
| CC26 | van Bemmel *et al.* 2013 | CG7357 |  | Set-N Chromatin Protein | AGAP004651, AGAP011262, AGAP010009 | -(other) | -(none) | - | - |
| CC27 | van Bemmel *et al.* 2013 | CG7928 |  | Set-N Chromatin Protein | AGAP002241 | + | -(none) | + | AGAP002241 |
| CC28 | van Bemmel *et al.* 2013 | CG4617 |  | Set-N Chromatin Protein | AGAP006392 | + | + | + | AGAP006392 |
| CC29 | van Bemmel *et al.* 2013 | CG33213 |  | Set-N Chromatin Protein | AGAP007915, AGAP006758 | -(other) | -(none) | - | - |
| CC3 | van Bemmel *et al.* 2013 | CG5245 |  | Set-N Chromatin Protein | AGAP007915, AGAP004651, AGAP007389 | -(other) | -(none) | - | - |
| CC30 | van Bemmel *et al.* 2013 | CG15514 |  | Set-N Chromatin Protein | AGAP009349 | + | -(none) | + | AGAP009349 |
| CC31 | van Bemmel *et al.* 2013 | CG9797 |  | Set-N Chromatin Protein | AGAP003224, AGAP011262 | -(other) | -(none) | - | - |
| CC32 (Zif) | van Bemmel *et al.* 2013 | CG10267 | Zif | Set-N Chromatin Protein | AGAP002918, AGAP011655, AGAP008845 | -(other) | -(none) | - | - |
| CC33 | van Bemmel *et al.* 2013 | CG4936 |  | Set-N Chromatin Protein | AGAP004651, AGAP007915, AGAP007389 | -(other) | -(none) | - | - |
| CC34 | van Bemmel *et al.* 2013 | CG10949 |  | Set-N Chromatin Protein | No Hits | -(other) | +(AGAP002753) | - | - |
| CC35 | van Bemmel *et al.* 2013 | CG7946 |  | Set-N Chromatin Protein | AGAP009140 | -(other) | +(AGAP008006) | - | - |
| CC4 | van Bemmel *et al.* 2013 | CG8289 |  | Set-N Chromatin Protein | AGAP003597, AGAP009444 | -(other) | -(none) | - | - |
| CC5 | van Bemmel *et al.* 2013 | CG7006 |  | Set-N Chromatin Protein | AGAP002330 | + | + | + | AGAP002330 |
| CC6 | van Bemmel *et al.* 2013 | CG15436 |  | Set-N Chromatin Protein | AGAP004651, AGAP007389, AGAP007915 | -(other) | -(none) | - | - |
| CC7 | van Bemmel *et al.* 2013 | CG17385 |  | Set-N Chromatin Protein | AGAP007389, AGAP004651 | -(other) | -(none) | - | - |
| CC8 | van Bemmel *et al.* 2013 | CG7745 |  | Set-N Chromatin Protein | AGAP006800, AGAP012074 | -(other) | -(none) | - | - |
| CC9 | van Bemmel *et al.* 2013 | CG8924 |  | Set-N Chromatin Protein | AGAP002303 | + | +(AGAP002303+  AGAP002304) | + | AGAP002303 |
| CDK7 | Filion *et al.* 2010 | CG3319 | Cyclin-dependent kinase 7 |  | AGAP002646 | + | + | + | AGAP002646 |
| Cfp1 | GO | CG17446 |  | Histone Methylation | AGAP004704 | + | + | + | AGAP004704 |
| CG10395 | GO | CG10395 |  | Ino80 Complex | AGAP006175 | + | + | + | AGAP006175 |
| CG11970 | GO | CG11970 |  | Ino80 Complex | AGAP012437 | - | - | - | - |
| CG12316 | GO | CG12316 |  | Histone Acetylation | No Hits | N/A | -(none) | - | - |
| CG18004 | GO | CG18004 |  | Ino80 Complex | AGAP001031 | + | -(none) | + | AGAP001031 |
| CG2051 | GO | CG2051 |  | Histone Acetylation | AGAP003264 | + | + | + | AGAP003264 |
| CG8677 | GO | CG8677 |  | RSF | AGAP001386 | + | + | + | AGAP001386 |
| Chm | Swaminathan *et al. 12* | CG5229 | chameau | Acetyltransferase | AGAP009676 | + | + | + | AGAP009676 |
| Chrac-14 | GO | CG13399 | Chromatin accessibility complex 14kD protein | Chrac | AGAP012825, AGAP005051 | ++ | ++ | ++ | AGAP012825, AGAP005051 |
| Chrac-16 | GO | CG15736 | Chromatin accessibility complex 16kd protein | Chrac | AGAP007481 | + | + | + | AGAP007481 |
| CoRest | GO | CG42687 |  | Histone Methylation/Regulation of Acetylation | AGAP002488 | + | + | + | AGAP002488 |
| CtBP | Filion *et al.* 2010 | CG7583 | C-terminal Binding Protein |  | AGAP003893 | + | + | + | AGAP003893 |
| CTCF | Filion *et al.* 2010 | CG8591 |  |  | AGAP005555 | + | + | + | AGAP005555 |
| CYP33 | van Bemmel *et al.* 2013 | CG4886 | cyclophilin-33 | Set-N Chromatin Protein | AGAP009810 | + | + | + | AGAP009810 |
| D1 | Filion *et al.* 2010 | CG9745 | D1 chromosomal protein |  | No Hits | N/A | -(none) | - | - |
| DF31 | Filion *et al.* 2010 | CG2207 | Decondensation factor 31 |  | No Hits | N/A | -(none) | - | - |
| Dik | GO | CG7098 | diskette | Histone Acetylation/phosphorylation | AGAP010459 | + | + | + | AGAP010459 |
| DMAP1 | GO | CG11132 |  | Histone Acetylation | AGAP007387 | + | + | + | AGAP007387 |
| Dp1 | GO | CG5170 | Dodeca-satellite-binding protein 1 | Heterochromatin | AGAP005467 | + | + | + | AGAP005467 |
| DSP1 | Filion *et al.* 2010 | CG12223 | dorsal switch protein 1 |  | AGAP000005 | + | + | + | AGAP000005 |
| DWG | Filion *et al.* 2010 | CG2711 | deformed wings |  | AGAP007389, AGAP004651 | - | -(none) | - | - |
| E(bx) | GO | CG32346 | enhancer of bithorax | NURF | AGAP006133 | + | + | + | AGAP006133 |
| E(Pc) | GO | CG7776 | enhancer of polcomb | Intercalary Heterochromatin | AGAP008026 | + | + | + | AGAP008026 |
| e(y)3 | GO | CG12238 | enhancer of yellow 3 | Heterochromatin | AGAP001877 | + | -(none) | + (AGAP001875) | - |
| Eaf6 | GO | CG12756 |  | NuA4 | AGAP006810 | + | + | + | AGAP006810 |
| ebi | GO | CG4063 |  | Histone Regulation of Deactylation | AGAP007739 | + | + | + | AGAP007739 |
| ECR | Filion *et al.* 2010 | CG1765 | ecdysone receptor |  | AGAP012211 | + | + | + | AGAP012211 |
| EFF | Filion *et al.* 2010 | CG7425 | effete |  | AGAP000145 | + | + | + | AGAP000145 |
| egg | GO | CG12196 | eggless | Histone Methylation | AGAP007978 | + | + | + | AGAP007978 |
| Esc | GO | CG14941 | Extra Sexcombs | Histone Methylation | AGAP008550 | + | + | + | AGAP008550 |
| Escl | GO | CG5202 |  | Histone Methylation | AGAP008550 | + | + | - | AGAP008550 |
| EZ | GO | CG6502 | Enhancer of zeste | Histone Methylation | AGAP012516 | + | + | + | AGAP012516 |
| FK506-bp1 | GO | CG6226 | FK506-binding protein 1 | histone peptidyl-prolyl isomerization | AGAP007472 | - | +(AGAP007473) | +(AGAP007473) | AGAP007473 |
| G9a | GO | CG2995 |  | Histone Methylation | AGAP003013 | + | + | + | AGAP003013 |
| Gcn5 | GO | CG4107 | Gcn5 ortholog | Histone Acetylation | AGAP004434 | + | + | + | AGAP004434 |
| gpp | Greer *et al. 12* | CG42803 | grappa | METHYLATION | AGAP003282 | + | + | + | AGAP003282 |
| GRO | Filion *et al.* 2010 | CG8384 | groucho |  | AGAP010324, AGAP004280 | ++ | ++ | ++ | AGAP010324, AGAP004280 |
| Hcf | GO | CG1710 | Host cell facter | Histone Methylation/Acetylation | AGAP004774 | + | + | + | AGAP004774 |
| Hdac3 | GO | CG2128 | Histone Deacetylase 3 | Histone Deacetylation | AGAP001143 | + | + | + | AGAP001143 |
| HDAC4 | Swaminathan *et al. 12* | CG1770 |  | Histone Deacetylation | AGAP000410 | + | + | + | AGAP000410 |
| HDAC6 | GO | CG6170 |  | Histone Deacetylation | AGAP000532 | + | + | + | AGAP000532 |
| HdacX | GO | CG31119 | Histone Deacetylase X | Histone Deacetylation | AGAP001736 | + | +(AGAP001736,  AGAP001737) | + | AGAP001736 |
| Hmt4-20 | GO | CG13363 | Histone methyltransferase 4-20 | Histone Methylation | AGAP000042 | + | + | + | AGAP000042 |
| HP1 | GO | CG8409 | Suppressor of variegation 205 | Histone/DNA Methylation/Centromeric + Nuclear HC | AGAP009444 | + | ++(AGAP009444, AGAP004723) | + | AGAP009444 |
| HP1b | GO | CG7041 | Heterochromatin Protein 1b | Heterochromatin | AGAP009444 | - | ++(AGAP009444, AGAP004723) | - | - |
| HP1c | GO | CG6990 | Heterochromatin Protein 1c | Heterochromatin | AGAP004723 | + | ++(AGAP009444, AGAP004723) | + | AGAP004723 |
| HP1e | GO | CG8120 | Heterochromatin Protein 1e | Heterochromatin | AGAP009444, AGAP004723 | - | -(none) | +(AGAP004723) | AGAP004723 |
| HP4 | GO | CG8044 | Heterochromatin Protein 4 | Heterochromatin | AGAP005561 | - | -(none) | - | - |
| HP5 | GO | CG1745 | Heterochromatin Protein 5 | Heterochromatin | No Hits | N/A | -(none) | - | - |
| HP6 | GO | CG15636 | Heterochromatin Protein 6 | Heterochromatin | AGAP004723, AGAP009444 | - | -(none) | +(AGAP004723) | AGAP004723 |
| Incenp | GO | CG12165 | Inner centromere protein | Histone phosphorylation | No Hits | N/A | -(none) | - | - |
| Ino80 | GO | CG31212 |  | Ino80 Complex | AGAP005035 | + | + | + | AGAP005035 |
| Ipod | GO | CG2961 | Interaction partner of Dnmt2 | DNA methylation | No Hits | N/A | -(none) | - | - |
| Iswi | GO | CG8625 | imitation SWI | ACF Complex | AGAP010700 | + | + | + | AGAP010700 |
| JHDM2 | GO | CG8165 | JmjC domain containing histone demethylase 2 | Histone Demethylation | AGAP002682 | + | + | + | AGAP002682 |
| JIGR2 | van Bemmel *et al.* 2013 | CG17383 | jing interacting gene regulatory 1 | Set-N Chromatin Protein | AGAP006800 | + | + | - | AGAP006800 |
| JIL-1 | GO | CG6297 |  | Histone phosphorylation | AGAP006094 | + | + | + | AGAP006094 |
| JRA | Filion *et al.* 2010 | CG2275 | Jun-related antigen |  | AGAP006386 | + | + | + | AGAP006386 |
| Kdm2 | GO | CG11033 | Lysince (K)-specific demethylase 2 | Histone Demethylation/Ubiquitination | AGAP001895 | + | + | + | AGAP001895 |
| Kdm4A | GO | CG15835 | Histone demethylase 4A | Histone Demethylation | AGAP011180 | + | + | + | AGAP011180 |
| Kdm4B | GO | CG33182 | Histone demethylase 4B | Histone Demethylation | AGAP006770 | + | + | + | AGAP006770 |
| LAM | Filion *et al.* 2010 | CG6944 | Lamin |  | AGAP008015, AGAP011938 | ++ | ++ | ++ | AGAP008015, AGAP011938 |
| Lhr | GO | CG18468 | lethal hybrid rescue | Heterochromatin | No Hits | N/A | -(none) | - | - |
| Lid | GO | CG9088 | Little imaginal discs | Histone Demethylation/Acetylation | AGAP004854 | + | + | + | AGAP004854 |
| LKR | GO | CG7144 | Lysine Ketoglutarate Reductase | Histone Methylation | AGAP008632 | + | + | + | AGAP008632 |
| LOLAL | Filion *et al.* 2010 | CG5738 | lola like |  | AGAP011247 | + | + | + | AGAP011247 |
| Lpt | GO | CG5591 | Lost PHDs of trr | Histone Methylation | 3L(18890039-18892840) | + | - | - | - |
| MAF-S | van Bemmel *et al.* 2013 | CG9954 |  | Set-N Chromatin Protein | AGAP010405 | + | + | + | AGAP010405 |
| MAX | Filion *et al.* 2010 | CG9648 |  |  | AGAP003177 | + | -(none) | + | AGAP003177 |
| MBD-like | GO | CG8208 |  | NuRD Complex | AGAP003982 | + | + | + | AGAP003982 |
| Mdg4 | Schulze *et al. 06* | CG32491 | modifier of mdg4 | Heterochromatin | AGAP003439 | + | - | + | AGAP003439 |
| MED31 | Filion *et al.* 2010 | CG1057 | Mediator complex subunit 31 |  | AGAP002258 | + | + | + | AGAP002258 |
| MEP-1 | GO | CG1244 |  | NuRD Complex | AGAP006601 | + | + | + | AGAP006601 |
| Mes-4 | Swaminathan *et al. 12* | CG4976 |  | Dimethylation | AGAP004656 | + | + | + | AGAP004656 |
| Mi-2 | GO | CG8103 |  | NuRD Complex | AGAP012009 | + | + | + | AGAP012009 |
| MLE | Swaminathan *et al. 12* | CG11680 | maleless | Acetylation activity | AGAP008239,  AGAP002223 | - | +(AGAP006599) | +(AGAP002223, AGAP006599, AGAP008239) | AGAP006599 |
| Mnn1 | GO | CG13778 | Menin 1 | Histone Methylation | AGAP008130 | + | + | + | AGAP008130 |
| MNT | Filion *et al.* 2010 | CG13316 |  |  | AGAP013301 | + | + | +(AGAP003177) | AGAP013301 |
| Mof | GO | CG3025 | Males absent on the first | Histone Acetylation | AGAP012229 | + | + | + | AGAP012229 |
| MRG15 | GO | CG6363 |  | Histone Acetylation | AGAP001795 | + | + | + | AGAP001795 |
| msl-1 | GO | CG10385 | male specific lethal 1 | Histone Acetylation | No Hits | N/A | -(none) | - | - |
| MSL-3 | Swaminathan *et al. 12* | CG8631 | male specific lethal 3 | Acetylation activity | AGAP001917 | + | + | + | AGAP001917 |
| Mt2 | GO | CG10692 | Methyltransferase 2 | DNA methylation | AGAP004101, AGAP012836 | ++ | ++ | ++ | AGAP004101, AGAP012836 |
| MUS209 | Filion *et al.* 2010 | CG9193 | mutagen-sensitive 209 |  | AGAP010220 | + | + | + | AGAP010220 |
| Nap1 | GO | CG5330 | nucleosome assembly protein 1 | ACF Complex | AGAP001928 | + | + | ++(AGAP009693) | AGAP001928 |
| NC2beta | GO | CG4185 | Negative cofactor 2beta | Histone Acetylation | AGAP010322 | + | + | + | AGAP010322 |
| Ncoa6 | GO | CG14023 |  | Histone Methylation | AGAP007771 | + | -(none) | + | AGAP007771 |
| neb | GO | CG10718 | nebbish | Heterochromatin | AGAP008035 | + | + | + | AGAP008035 |
| nej | GO | CG15319 | nejire | Histone Acetylation | AGAP000029 | + | + | + | AGAP000029 |
| not | GO | CG4166 | non-stop | Histone Deubiquitination | AGAP012119 | + | + | + | AGAP012119 |
| Nurf-38 | GO | CG4634 | nucleosome remodeling factor - 38kD | NURF | AGAP003398 | + | + | + | AGAP003398 |
| Orc2 | GO | CG3041 | Origin recognition complex subunit 2 | Alpha-Heterochromatin | AGAP000474 | + | + | + | AGAP000474 |
| Pa1 | GO | CG11750 | PTIP associated 1 | Histone Methylation | AGAP003353 | + | + | + | AGAP003353 |
| Parg | GO | CG2864 | Poly(ADP-ribose) glycohydrolase | Histone Regulation of Acetylation | AGAP000589 | + | + | -(AGAP003831) | AGAP000589 |
| Pc | GO | CG32443 | polycomb | Intercalary Heterochromatin | 2L (26898592-2757082) | + |  | - | - |
| PCL | Filion *et al.* 2010 | CG13374 | pepsinogen-like |  | AGAP003277 | -(other) | -(none) | + | - |
| pho | GO | CG17743 | pleiohomeotic | Ino80 Complex | AGAP009348 | -(other) | - | - | - |
| PHOL | Filion *et al.* 2010 | CG3445 | pleiohomeotic like |  | AGAP009348, AGAP006592 | -(other) | -(none) | - | - |
| piwi | GO | CG6122 |  | Telomeric Heterochromatin | AGAP009509, AGAP011204 | ++ | ++ | ++ | AGAP009509, AGAP011204 |
| pont | GO | CG4003 | pontin | Ino80 Complex | AGAP009746 | - (2nd) | -(none) | + | AGAP009746 |
| Pr-set7 | GO | CG3307 |  | Histone Methylation/Regulation of Acetylation | AGAP012481 | + | +(AGAP012481, AGAP012610) | + | AGAP012481 |
| prod | GO | CG18608 | proliferation disrupter | Centromeric Heterochromatin | No Hits | N/A | -(none) | - | - |
| Psc | GO | CG3886 | posterior sex combs | Intercalary Heterochromatin | AGAP006403 | + | + | + | AGAP006403 |
| Ptip | GO | CG32133 |  | Histone Methylation | AGAP001710 | + | + | +(AGAP001709) | AGA001709,AGAP001710 |
| pzg | GO | CG7752 | putzig | NURF | AGAP006534 | + | + | - | AGAP006534 |
| Rbbp5 | GO | CG5585 |  | Histone Methylation | AGAP010575 | + | +(AGAP010574, AGAP010575) | + | AGAP010575 |
| rept | GO | CG9750 | reptin | Ino80 Complex | AGAP009746 | + | + | + | AGAP009746 |
| rhi | GO | CG10683 | rhino | Heterochromatin | AGAP009444 | - | -(none) | +(AGAP009444) | AGAP009444 |
| Rif1 | GO | CG30085 | Rap1 interacting factor 1 homolog | Nuclear Heterochromatin | AGAP010393 | + | + | + | AGAP010393 |
| Rpb4 | GO | CG43662 |  | Histone Acetylation | AGAP001228 | + | -(none) | + | AGAP001228 |
| Rpd3 | GO | CG7471 |  | NuRD Complex | AGAP006511 | + | + | + | AGAP006511 |
| RPII18 | Filion *et al.* 2010 | CG1163 | RNA polymerase II 18kD subunit |  | AGAP005873 | + | + | + | AGAP005873 |
| Rtf1 | GO | CG10955 |  | Histone Methylation | AGAP012057 | + | + | + | AGAP012057 |
| SA | GO | CG3423 | stromalin | Centromeric Heterochromatin | AGAP006998 | + | + | + | AGAP006998 |
| SBP2 | van Bemmel *et al.* 2013 | CG7066 | SECIS-binding protein 2 | Set-N Chromatin Protein | AGAP002276 | + | + | + | AGAP002276 |
| Sce | GO | CG5595 | sex combs extra | Histone Ubiquitination | AGAP002073 | + | + | + | AGAP002073 |
| scny | GO | CG5505 | scrawny | Histone Deubiquitination | AGAP007521 | + | + | + | AGAP007521 |
| Set1 | GO | CG40351 |  | Histone Methylation | AGAP002246 | + | + | + | AGAP002246 |
| Set2 | GO | CG1716 |  | Histone Methylation | AGAP011688 | + | + | + | AGAP011688 |
| Sgf29 | GO | CG30390 | SAGA-associated factor 29 ortholog | Histone Acetylation | AGAP005116 | + | + | + | AGAP005116 |
| simj | GO | CG32067 | simjang | NuRD Complex | AGAP005619 | + | + | + | AGAP005619 |
| Sin3 | Swaminathan *et al. 12* | CG8815 | Sin3A | Histone Deacetylation Corepressor | AGAP007892 | + | + | + | AGAP007892 |
| Sir2 | GO | CG5216 |  | Histone Deactylation | AGAP002943 | - | -(none) | - | AGAP002943 |
| SSP | van Bemmel *et al.* 2013 | CG17153 | sunspot | Set-N Chromatin Protein | AGAP005746 | -(other) | -(none) | - | - |
| SU(VAR)2-10 | Filion *et al.* 2010 | CG8068 | Suppressor of variegation 2-10 |  | AGAP005031 | + | + | + | AGAP005031 |
| Su(var)2-HP2 | GO | CG12864 |  | Nuclear Heterochromatin | No Hits | N/A | -(none) | +(AGAP001194) | - |
| Su(var)3-3 | GO | CG17149 | supressor of variegation 3-3 | Histone Demethylation | AGAP011661 | + | + | + | AGAP011661 |
| Su(var)3-7 | GO | CG8599 | Suppressor of variegation 3-7 | Centromeric Heterochromatin | No Hits | N/A | -(none) | - | - |
| Su(var)3-9 | GO | CG43664 | Suppressor of variegation 3-9 | Histone/DNA Methylation/HC | AGAP003597 | - | + | - | AGAP003597 |
| Su(z)12 | GO | CG8013 |  | Histone Methylation | AGAP011881 | + | + | + | AGAP011881 |
| Su(z)2 | GO | CG3905 | suppressor of zeste 2 | Intercalary Heterochromatin | AGAP006402 | + | -(none) | + | AGAP006402 |
| SuUR | GO | CG7869 | Suppressor of Under-replication | Centromeric/Telomeric/Intercalary Heterochromatin | AGAP005819 | + | -(none) | + | AGAP005819 |
| Taf1 | Swaminathan *et al. 12* | CG17603 | TBP-associated factor 1 | Acetyltransferase/Ubiquitinase | AGAP003882 | + | + | + | AGAP003882 |
| TBP | Filion *et al.* 2010 | CG9874 | TATA binding protein |  | AGAP008084 | + | +(AGAP010958) | +(AGAP010958) | AGAP010958 |
| tefu | GO | CG6535 | telomere fusion | Histone phosphorylation | AGAP009632 | + | + | + | AGAP009632 |
| Tip60 | GO | CG6121 |  | Histone Acetylation | AGAP001539 | + | + | + | AGAP001539 |
| tlk | GO | CG34412 | Tousled-like kinase | Histone phosphorylation | AGAP000043 | + | + | + | AGAP000043 |
| TOP1 | Filion *et al.* 2010 | CG6146 | Topoisomerase 1 |  | AGAP004078 | + | + | + | AGAP004078 |
| TRIP1 | van Bemmel *et al.* 2013 | CG8882 |  | Set-N Chromatin Protein | AGAP006607 | + | + | + | AGAP006607 |
| Trl | GO | CG33261 | Trithorax-like | Centromeric Heterochromatin | AGAP011583 | + | +(AGAP011582, AGAP011583) | + | AGAP011583 |
| Trr | GO | CG3848 | Trithorax-related | Histone Methylation | AGAP011192 | + | + | + | AGAP011192 |
| Trx | GO | CG8651 | Trithorax | Histone Methylation/Acetylation | AGAP002741 | + | + | + | AGAP002741 |
| Usp7 | GO | CG1490 | ubiquitin-specific protease 7 | Histone Deubiquitination | AGAP008530 | + | + | + | AGAP008530 |
| Utx | GO | CG5640 |  | Histone Demethylation | AGAP008509 | + | + | + | AGAP008509 |
| Vig2 | GO | CG11844 |  | Histone Methylation | No Hits | N/A | +(AGAP013112) | - | - |
| vtd | GO | CG17436 | verthandi | Centromeric Heterochromatin | AGAP004560 | + | + | + | AGAP004560 |
| wda | GO | CG4448 | will decrease acetylation | Histone Acetylation | AGAP003524 | + | + | + | AGAP003524 |
| Wdr82 | GO | CG17293 |  | Histone Methylation | AGAP009700 | + | + | + | AGAP009700 |
| Wds | GO | CG17437 | Will die slowly | Histone Methylation/Acetylation | AGAP002019, AGAP012731 | ++ | ++ | ++ | AGAP002019, AGAP012731 |
| woc | GO | CG5965 | without children | Heterochromatin | AGAP001140 | + | + | + | AGAP001140 |
| XNP | GO | CG4548 |  | Beta-Heterochromatin | AGAP002490 | + | + | +(AGAP002490, AGAP009344) | AGAP002490 |
| YL-1 | GO | CG4621 |  | Histone Acetylation | AGAP001565 | + | + | - | AGAP001565 |
| Yp2 | GO | CG2979 | Yolk protein 2 | ACF Complex | AGAP009101, AGAP011682 | - | -(none) | - | - |
| z | GO | CG7803 | zeste | Intercalary Heterochromatin | No Hits | N/A | -(none) | - | - |

1. Indicates gene name in *Drosophila melanogaster*  according to <http://www.flybase.org> [↑](#footnote-ref-1)
2. Respective source of gene that attributes the gene to an epigenetic process [↑](#footnote-ref-2)
3. Description of gene in http://flybase.org [↑](#footnote-ref-3)
4. Description of epigenetic function that the gene is involved within [↑](#footnote-ref-4)
5. Results of BLAST against *Anopheles gambiae* genome. “No Hits” indicate no hits found during blast [↑](#footnote-ref-5)
6. Results of reciprocal best BLAST of *An. gambiae* BLAST results against *D. melanogaster* genome. “+” indicates RBB yielded original *D.*melanogaster gene as the top hit while “-“ indicates it was not the top RBB hit and between paraenthesis is the result of the original *D. melanogaster* gene if not RBB top hit. [↑](#footnote-ref-6)
7. OrthoDB’s orthologous call in *An. gambiae*, “+” indicates result agrees with BLAST, “-“ indicates a call not concurrent with BLAST hit with description of call in parenthesis [↑](#footnote-ref-7)
8. eggNog’s orthologous call in *An. gambiae*, “+” indicates result agrees with BLAST, “-“ indicates a call not concurrent with BLAST hit with description of call in parenthesis [↑](#footnote-ref-8)
9. Final *An. gambiae* orthologous gene call to the *D. melanogaster* gene in the first column. “-“ indicates no orthologous gene has been identified in this study. All genes in these columns are presented as AGAP identifiers from http://www.vectorbase.org [↑](#footnote-ref-9)
